# Safety concerns associated with high-dose continuous infusion of cefepime among critically ill patients with mild renal impairment to augmented renal clearance at a level 1 trauma center: CEFTOX study

**DOI:** 10.1128/aac.01735-25

**Published:** 2026-02-12

**Authors:** Myriam Lamamri, Charlotte Rutman, Anais Codorniu, Mathilde Holleville, Stéphanie Sigaut, Emmanuel Weiss, Caroline Jeantrelle

**Affiliations:** 1 Département d'Anesthésie Réanimation, AP-HP, Hôpital Beaujon, DMU PARABOL 55100, Clichy, France; 2 NeuroDiderot, Neuroprotection of the Developing Brain, Université Paris Cité, INSERM UMR-S 1141 555089, Paris, France; 3 Centre de Recherche sur l'Inflammation, Inserm UMR_S 1149 et Université Paris Cité 555089, Paris, France; University of Houston, Houston, Texas, USA

**Keywords:** augmented renal clearance, cefepime threshold, cefepime-induced neurotoxicity, pharmacokinetic, therapeutic drug monitoring

## Abstract

High-dose continuous infusion of cefepime is frequently employed in ICU patients with a creatinine clearance above 60 mL/min. The CEFTOX study aimed to investigate whether this regimen could lead to cefepime overexposure and cefepime-induced neurotoxicity (CIN) in a cohort of severe trauma and brain-injured patients. This retrospective cohort study included patients from a Level 1 Trauma Center who received a continuous infusion of 6 g/day of cefepime and had a therapeutic drug monitoring (TDM) within 24–48 h of treatment initiation. They were divided into three groups based on creatinine clearance: mild renal impairment (60–90 mL/min), normal clearance (90–150 mL/min), and augmented renal clearance (ARC) (>150 mL/min). The primary outcome was cefepime overexposure. A key secondary outcome was CIN. One hundred and sixty-two critically ill patients were included: 84 with ARC, 62 with normal renal clearance, and 16 with mild renal impairment. Cefepime overexposure occurred in 72 (44.4%) patients. While 50% of patients with normal renal clearance experienced overexposure, the rate was higher in those with mild renal impairment (87.5%) and lower in those with ARC (32.1%; *P* < 0.0001). In the ARC group, age > 33 years was a risk factor for overexposure (odds ratio [OR] 3.76; 95% CI [1.30–10.95]; *P* = 0.01), while sepsis was a protective factor (OR 0.30; 95% CI [0.11–0.83]; *P* = 0.02). CIN was observed in 24% of overexposed patients when TDM results were obtained ≤48 h compared to 57.4% when results were delayed >48 h (*P* = 0.006). These results highlight the need for early TDM and individualized dose adjustment to avoid CIN.

## INTRODUCTION

Cefepime, a fourth-generation cephalosporin, is a cornerstone for empirical treatment of severe infections in critically ill patients due to its broad spectrum and stability against β-lactamases (1, 2). For optimal efficacy, pharmacokinetic/pharmacodynamic (PK/PD) target recommends an unbound serum concentration at least 4 times above the minimum inhibitory concentration (MIC) throughout the dosing interval (i.e., achieving 100% *f*T > 4× MIC) (3, 4). To achieve this goal, continuous high-dose regimens frequently reaching 6 g per day are commonly used in patients with severe infections and creatinine clearance (CrCl) above 60 mL/min, especially during empirical treatment targeting potentially resistant pathogens (1, 5).

However, maintaining efficacy must be balanced against safety. Supra-therapeutic cefepime concentrations have been reported in up to 80.7% of medical ICU patients receiving these high doses (6). Furthermore, cefepime-induced neurotoxicity (CIN) affects approximately 15% of medical ICU patients and may necessitate interventions like antiepileptic drugs or dialysis (7). The main reported risk factors for CIN include the following: high cefepime concentrations with thresholds varying according to the infusion method (continuous or intermittent) and patient population, pre-existing brain injury, advanced age, and renal impairment (8–11). While dose reduction is standard for patients with CrCl < 60 mL/min, CIN has still been reported in 26% of patients receiving renally adjusted doses (12).

Augmented renal clearance (ARC) is highly prevalent among young trauma and brain-injured patients, raising concerns about underexposure and treatment failure (13–15). Consequently, high-dose continuous infusion has been justified as a strategy to minimize underdosing. Yet, given cefepime’s narrow therapeutic window, it remains unclear whether this approach inadvertently increases the risk of a steady-state cefepime concentrations above 35 mg/L, a reported threshold associated with CIN during continuous infusion (4, 16).

Therefore, we aimed to leverage our routine therapeutic drug monitoring (TDM) data to determine the prevalence of cefepime steady-state plasma concentration exceeding 35 mg/L in trauma and brain-injured patients receiving a 6 g/day continuous infusion according to their creatinine clearance. Secondary objectives included the following: assessing the prevalence of CIN and defining a threshold associated with CIN in a vulnerable population of patients with acute brain injury; identifying risk factors for both overexposure and CIN specifically in the ARC group; and comparing the incidence of CIN between patients who received a dose adjustment guided by TDM results and those who did not.

## MATERIALS AND METHODS

### Study design

We performed a retrospective, single-center, observational study based on electronic medical records. This study is reported in accordance with the Strengthening the Reporting of Observational Studies in Epidemiology (STROBE) statement (17).

### Population and cefepime TDM

Patients diagnosed with an infection during their ICU stay, between 1 May 2021 and 31 December 2024, were screened using validated International Classification of Diseases Codes. Our local cefepime initiation protocol, in line with the rationale for high-dose regimens in severe infections, started on 1 May 2021. Patients were eligible if they had (i) a documented infection requiring cefepime therapy initiated at 6 g/day by continuous infusion following a 2 g loading dose, (ii) a CrCl above 60 mL/min, and (iii) simultaneous TDM and 24-h creatinine clearance assessment performed between 24 and 48 h after cefepime initiation. Exclusion criteria were the absence of TDM or creatinine clearance measurement.

The total cefepime plasma steady-state concentration was quantified using high-performance liquid chromatography. Details of the method of the cefepime assay are provided in the Supplemental methods. According to our protocol, the cefepime dose should be adjusted based on TDM results. However, TDM is only performed twice weekly (Tuesdays and Fridays) during the day in our center.

### Definitions

CrCl (in mL/min), based on a 24-h urine collection, was calculated daily, concurrently with TDM, using the UV/P formula, where U represents the urine creatinine concentration (mmol/L), V represents the 24-h urine volume (liter), and P represents the plasma creatinine concentration (µmol/L). The 24-h creatinine clearance assessment was a standard of care in our center.

Patients receiving continuous high doses of cefepime were stratified into three groups using 24-h unindexed creatinine clearance values, determined by the UV/P formula:

- Mild renal impairment: between 60 and 90 mL/min
- Normal renal clearance: between 90 and 150 mL/min
- Augmented renal clearance: above 150 mL/min. The threshold for ARC was set at >150 mL/min, a value associated with significant cephalosporin clearance variations in critically ill populations and known to have a clinical impact on patients, such as drug underexposure and subsequent treatment failure in the ICU (18).

Furthermore, creatinine clearance indexed to body surface area using the Dubois formula was calculated as an additional parameter to the unindexed value (19).

Sepsis and septic shock were defined according to the Sepsis-3 definition (20).

### Outcomes

The primary outcome was cefepime overexposure, defined as a cefepime steady-state plasma concentration exceeding 35 mg/L. This threshold was selected because it is reported to be a potential toxicity threshold that correlates with an increased risk of CIN (16). For this reason, it is recommended by the French Society of Anesthesia, Critical Care, and the French Society of Pharmacology, which specifically advise that, during continuous infusion, the cefepime steady-state plasma concentration of 35 mg/L should not be exceeded (4). Furthermore, the 35 mg/L cutoff aligns with our local institutional protocol, which uses this concentration to flag overexposure and mandate dose adjustments.

Secondary outcomes were as follows:

- CIN was assessed by two independent neuro-intensivists with over 10 years of clinical experience (including one with specialized training in electroencephalogram interpretation). They independently reviewed patient medical records to establish the diagnosis of CIN blinded to the steady-state cefepime concentration results of each patient.

Consistent neurological signs and/or symptoms were graded according to the National Cancer Institute Common Terminology Criteria for Adverse Events (NCIC), to identify CIN (21). Neurological symptoms or signs were attributed to CIN only after excluding all other possible etiologies. The two neuro-intensivists systematically assessed the occurrence of all potential drug-related adverse events. Causality was assessed using the WHO-UMC (Uppsala Monitoring Centre) system for standardized case causality assessment. Only manifestations classified as “certain” or “probable/likely” were retained as being related to CIN (22). Patients who were clinically unevaluable due to profound sedation with a RASS sedation score of −4 to −5 (i.e., under profound general anesthesia) were excluded from the CIN analysis (23).

An EEG pattern consistent with cefepime toxicity was defined as the presence of triphasic waves, nonconvulsive status epilepticus, areactive pseudoperiodic or pseudorhythmic activities, lateralized rhythmic delta activity, or myoclonic status epilepticus (12). All EEGs were performed according to the International Federation of Clinical Neurophysiology guidelines and interpreted by an independent epileptologist (24). Routine EEG monitoring was not a standard practice in our center.

Following a review of the literature, we used an adapted CIN definition, whereby a patient was classified as CIN-positive if they met any one of the following criteria (10, 25–28): (i) they developed two or more new or worsening neurological symptoms (if the severity grade increased by at least one grade), that fulfilled the NCIC criteria, occurring 24 h after the initiation of cefepime treatment. (ii) If they had an EEG tracing consistent with neurotoxicity. (iii) If an improvement in symptoms was observed within 4 days of cefepime discontinuation, dose reduction, or initiation of a therapeutic intervention targeting the symptoms. Discrepancies were resolved by joint review and discussion by the two neuro-intensivists until a unanimous consensus was reached on the CIN classification.

- Delayed awakening was defined as the persistence of unresponsiveness to verbal commands for more than 5 days following cessation of sedation for a general anesthesia during cefepime therapy (29).
- Treatment failure was defined as the persistence or worsening of initial clinical and biological signs of infection at the end of the treatment course. Clinical criteria included assessment of temperature, vital signs, and signs and symptoms present at the time of infection diagnosis. Biological criteria included the persistence or worsening of leukocytosis or leukopenia. This outcome was assessed retrospectively by two study investigators based on patient medical records who had access to the TDM results.
- We retrospectively obtained the germ’s antibiogram and therefore used the EUCAST clinical breakpoint for susceptibility (C Susceptibility) (30). Thus, a patient with a steady-state cefepime concentration <4× C Susceptibility was classified as theoretically underexposed.

### Statistical analyses

Categorical variables are expressed as N (%), and continuous data are expressed as median (interquartile range [IQR]). Differences in baseline demographic, clinical, and infection-related characteristics across the three renal clearance groups were assessed using the Kruskal-Wallis test for continuous variables and the chi-square test or Fisher’s exact test (where appropriate) for categorical variables.

Factors associated with the cefepime overexposure and CIN were explored in univariable analysis using Student’s *t*-test or Wilcoxon test for continuous variables and chi-square test or Fisher’s exact test for categorical variables.

Clinical/demographic factors associated with a *P*-value of less than 0.2 in univariable analysis were included in a multivariable logistic regression model. The final selection was performed using the stepwise backward selection. Potential collinearity between variables was checked, and the more clinically relevant variable was retained in the case of collinearity. Results of multivariable analysis are reported with adjusted odds ratios (OR) and their 95% confidence interval (CI). Significant continuous variables identified in the univariable analysis were dichotomized to optimize their sensitivity and specificity using the Youden index with the creation of receiver operating characteristic (ROC) curves. Also, the receiver operating characteristic (ROC) curve method was used to determine an optimal Youden cut-off value on the cefepime plasma concentration to distinguish patients with and without risk of CIN (for the brain-injured patients). A two-tailed *P*-value <0.05 was considered significant. Statistical analysis was performed using R software version 4.2.2 for Windows (R Foundation for Statistical Computing, Vienna, Austria).

## RESULTS

### Population

**Fig 1:**
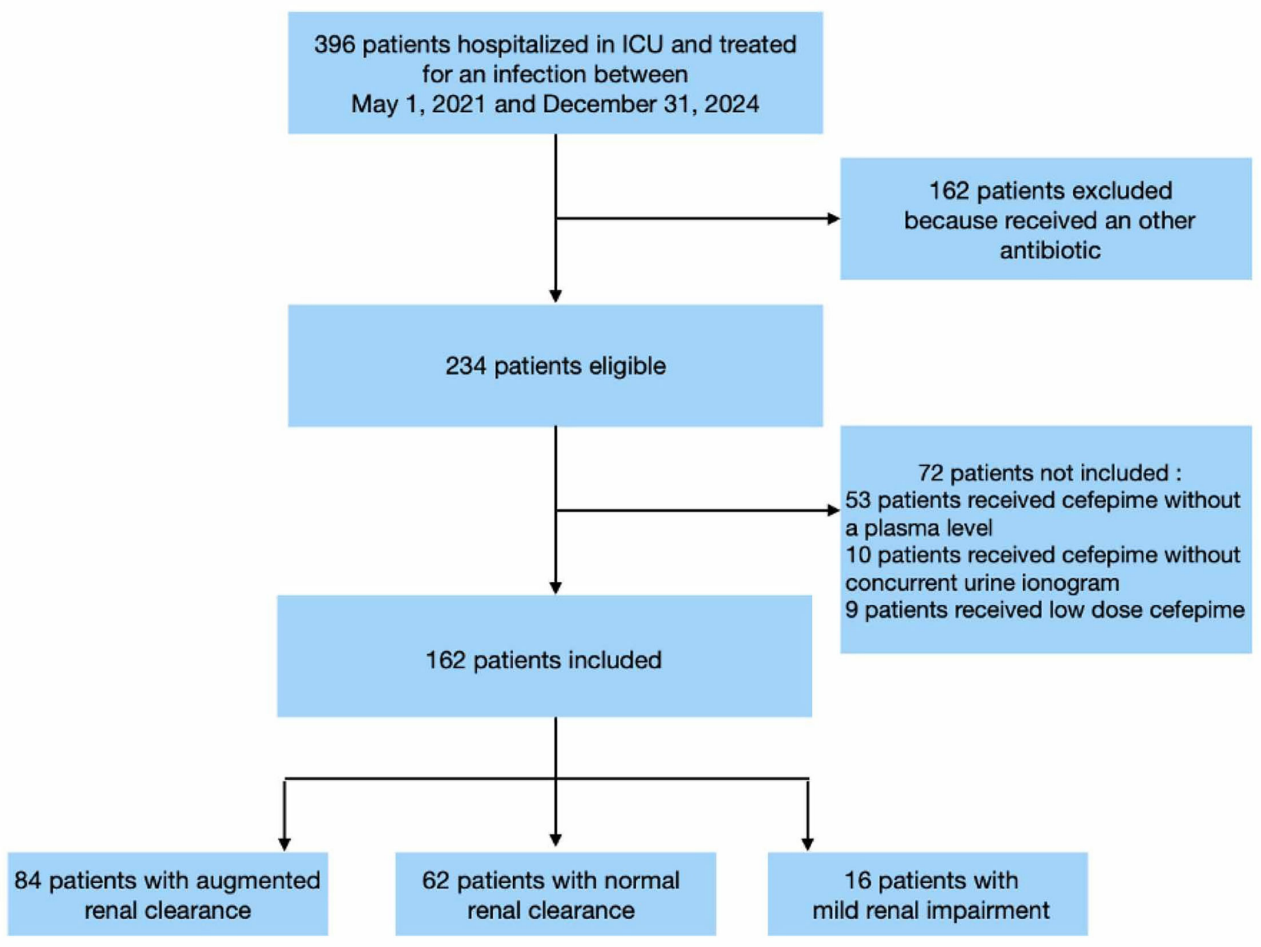
Flow chart.

Among the 396 patients hospitalized in the ICU during the study period who developed an infection, 234 were eligible and 162 patients were analyzed (Fig. 1). The baseline demographic, clinical, and infection-related characteristics of the patients are shown in Table 1. Causative pathogens are shown in Fig. S1.

**TABLE 1:** Baseline demographic, clinical, and infection-related characteristics^*a*^

Variables	Overall	60–90 mL/min	90–150 mL/min	>150 mL/min	*P*-value
	162 (100%)	16 (9.9%)	62 (38.3%)	84 (51.8%)	
Age (years); median (IQR)	44 [28–59]	69 [59–75]	47 [31–59]	36 [23–47]	< 0.0001
Male sex; *n* (%)	128 (79.0%)	12 (75.0%)	46 (74.2%)	70 (83.3%)	0.4
BMI (kg/m^2^); median (IQR)	25 [22.6–29.3]	30.5 [26.1–31.6]	24.5 [22.2–27.6]	24.8 [22.6–29.3]	0.03
ASA score, *n* (%)					0.001
1	65 (40.1%)	2 (12.5%)	22 (35.5%)	41 (48.8%)	
2	72 (44.4%)	8 (50.0%)	26 (41.9%)	38 (45.2%)	
3	25 (15.4%)	6 (37.5%)	14 (22.6%)	5 (6.0%)	
SAPS II; median (IQR)	46 [34–58]	53 [43.5–61.2]	45.5 [33–54]	45 [34–58]	0.4
SOFA score; median (IQR)	6 [4–8]	7 [4–10]	6 [4–8]	6 [4–7]	0.4
Reason for ICU admission; *n* (%)					<0.001
Severe trauma	137 (84.6%)	10 (62.5%)	51 (82.3%)	76 (90.5%)	
Hemorrhagic stroke	13 (8.0%)	4 (25.0%)	6 (9.7%)	3 (3.6%)	
Other neurological injuries	8 (4.9%)	0 (0%)	3 (4.8%)	5 (6.0%)	
Sepsis	4 (2.5%)	2 (12.5%)	2 (3.2%)	0 (0%)	
Traumatic brain injury; *n* (%)	109 (67.3%)	6 (37.5%)	38 (61.3%)	65 (77.4%)	<0.001
Hemorrhagic shock at admission; *n* (%)	36 (22.2%)	4 (25.0%)	19 (30.6%)	13 (15.5%)	0.08
Plasma creatinine at admission (µmol/L); median (IQR)	78 [60–99.8]	89.5 [73.8–119]	79 [63–99.8]	74 [58–95.5]	0.6
Site of infection, *n* (%)					0.3
Ventilator-associated pneumonia	127 (78.4%)	11 (68.8%)	49 (79%)	67 (79.8%)	
Limb surgical site infection	13 (8.0%)	2 (12.5%)	6 (9.7%)	5 (6.0%)	
Spine surgical site infection	9 (5.6%)	3 (18.8%)	2 (3.2%)	4 (4.8%)	
Meningitis	3 (1.9%)	0 (0%)	0 (0%)	3 (3.6%)	
Bacteremia	3 (1.9%)	0 (0%)	3 (4.8%)	0 (0%)	
Peritonitis	3 (1.9%)	0 (0%)	2 (3.2%)	1 (1.2%)	
Cerebral empyema	2 (1.2%)	0 (0%)	0 (0%)	2 (2.4%)	
Necrotizing fasciitis	1 (0.6%)	0 (0%)	0 (0%)	1 (1.2%)	
Prostatitis	1 (0.6%)	0 (0%)	0 (0%)	1 (1.2%)	
Empirically initiated cefepime; *n* (%)	130 (80.2%)	16 (100%)	51 (82.3%)	63 (75.0%)	0.06
Septic shock during the infection; *n* (%)	38 (23.5%)	7 (43.8%)	17 (27.4%)	14 (16.7%)	0.04
Sepsis during the infection; *n* (%)	96 (59.3%)	7 (43.8%)	38 (61.3%)	51 (60.7%)	0.4
Albuminemia at cefepime initiation (g/L); median (IQR)	22.4 [19.4–26.9]	20.8 [18–24.1]	21.6 [19–26]	23.3 [19.8–27.9]	0.07
Duration of cefepime treatment (days); median (IQR)	7 [4–7]	6 [4–7]	7 [4–7]	7 [4–7]	0.2
Fluid balance assessment at the initiation of cefepime (mL); median (IQR)	−275 [−1200 to 350]	−50 [−775 to 525]	−100 [−1025 to 425]	−572.5 [−1400 to 340]	0.2
Creatinine clearance concomitant with cefepime TDM (mL/min); median (IQR)	154 [121–198.5]	80 [72.2–85]	124.5 [114–135.5]	197 [167–229.2]	< 0.0001
Creatinine clearance using Dubois formula concomitant with cefepime TDM (mL/min/1.73 m^2^); median (IQR)	138 [107–173]	69 [59.8–70.5]	113 [99–127]	172.5 [148.8–206.8]	< 0.0001

^*a*^ N, number; IQR, interquartile range; BMI, body mass index; ASA, American Society of Anesthesiologists; ICU, intensive care unit; IQR, interquartile range; SAPS II, Simplified Acute Physiology Score II; SOFA, sequential organ failure assessment; TDM, therapeutic drug monitoring.

### Cefepime therapeutic drug monitoring

**Fig 2:**
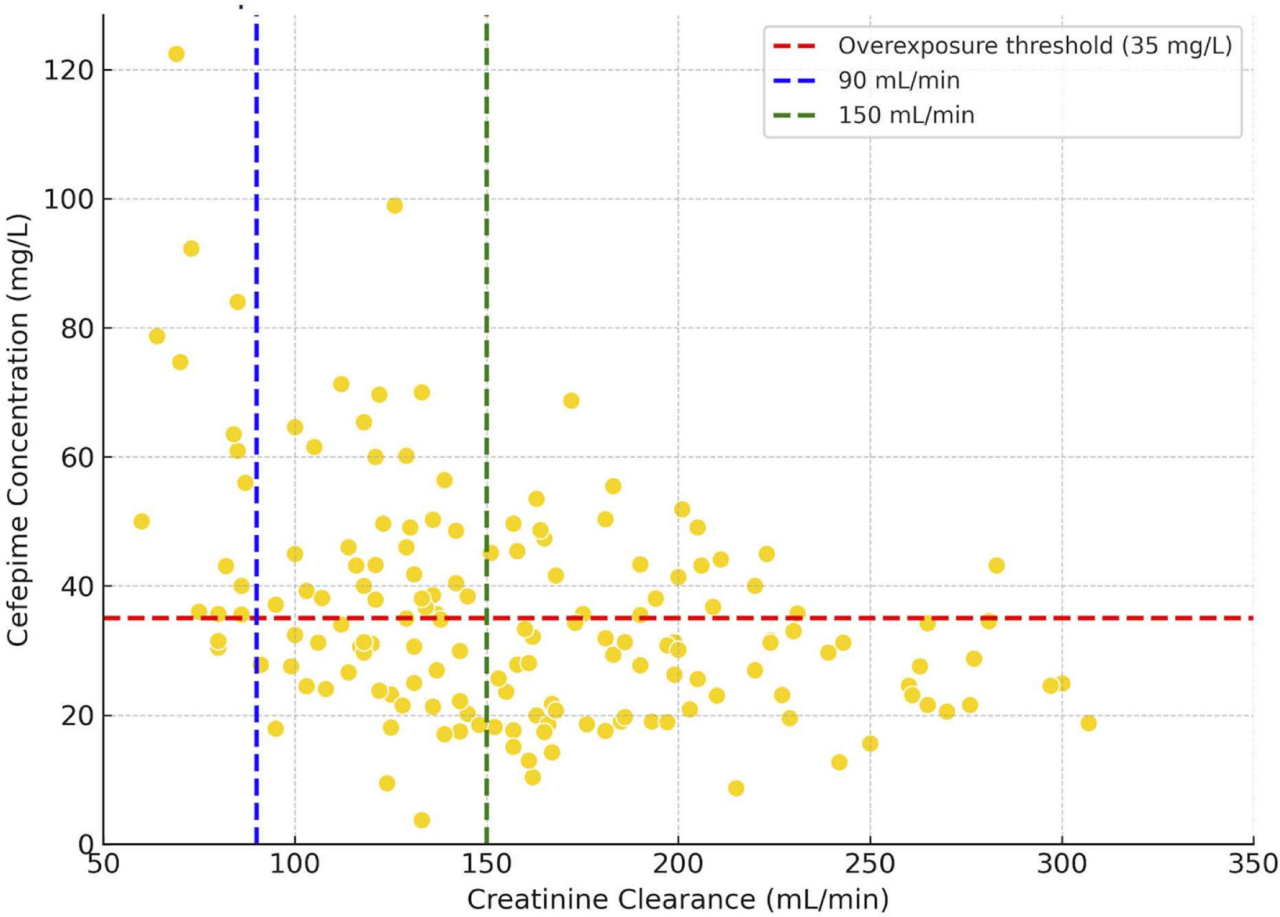
Scatter plot illustrating the relationship between creatinine clearance and cefepime plasma steady-state concentrations in our study population. Each yellow dot represents the dosage of a patient. The horizontal line (red line) indicates the overexposure threshold of 35 mg/L (cefepime plasma steady-state concentration above 35 mg/L). Each vertical line (blue and green) represents, respectively, the boundary between mild renal impairment and normal renal clearance, and the boundary between normal renal clearance and ARC.

The proportion of cefepime overexposure (> 35 mg/L) according to the creatinine clearance group is presented in Table 2. Overall, cefepime overexposure was identified in 72 (44.4%) patients. The prevalence differed significantly between groups (*P* < 0.0001): cefepime overexposure encountered in 87.5% of patients with mild renal impairment, 50% with normal renal clearance, and 32.1% with ARC. Individual patient plasma steady-state concentrations as a function of their creatinine clearance are shown in Fig. 2.

**TABLE 2:** Primary and secondary outcomes^*a*^

Variables	Overall	60–90 mL/min	90–150 mL/min	>150 mL/min	*P*-value
	162 (100%)	16 (9.9%)	62 (38.3%)	84 (51.8%)	
Primary outcome					
Cefepime overexposure (>35 mg/L); *n* (%)	72 (44.4%)	14 (87.5%)	31 (50.0%)	27 (32.1%)	<0.0001
Secondary outcomes					
Cefepime concentration >20 mg/L; *n* (%)	136 (84.0%)	16 (100%)	55 (88.7%)	65 (77.4%)	0.034
Cefepime plasma concentration (mg/L); median (IQR)	31.8 [23.3–43.3]	53 [35.9–75.7]	35.4 [25.4–45.8]	28.5 [20.7–36]	<0.0001
<4x C Susceptibility; *n* (%)	1 (0.6%)	0 (0%)	1 (3.0%)	0 (0%)	0.520
Not evaluable: under profound sedation; *n* (%)	41 (25.3%)	1 (6.3%)	13 (21.0%)	27 (32.1%)	0.060
Cefepime concentration >35 mg/L; *n* (%)^*b*^	13 (31.7%)	1 (100%)	3 (23.0%)	9 (33.3%)	0.453
Cefepime-induced neurotoxicity; *n* (%)	43 (36.1%)	9 (60%)	18 (36.7%)	16 (28.1%)	0.01
Delayed awakening during cefepime therapy; *n* (%)	17 (10.5%)	3 (18.8%)	10 (16.1%)	4 (4.8%)	0.031
EEG performed during cefepime therapy; *n* (%)	27 (16.7%)	7 (43.8%)	11 (17.7%)	9 (10.7%)	<0.001
Pathological EEG patterns^*c*^	8 (28.6%)	3 (42.9%)	3 (27.3%)	2 (20.0%)	0.667
Failure of infection treatment; *n* (%)	21 (13.0%)	3 (18.8%)	6 (9.7%)	12 (14.3%)	0.602
Dose adjustment; *n* (%)	30 (18.5%)	3 (18.7%)	10 (16.1%)	17 (20.2%)	0.832
Invasive mechanical ventilation, *n* (%)	158 (97.5%)	15 (93.7%)	62 (100%)	81 (96.4%)	0.229
Duration of mechanical ventilation (days); median (IQR) ^*d*^	17 [11–31]	18 [2–28]	16 [11–31]	17 [12–32]	0.576
ICU length of stay (days); median (IQR)	28 [18–41]	22.5 [14–31]	27 [18–41]	28 [20–45]	0.199
ICU mortality; *n* (%)	24 (14.8%)	3 (18.8%)	11 (17.7%)	10 (11.9%)	0.511

^*a*^ N, number; IQR, interquartile range; ICU, intensive care unit; EEG, electroencephalogram; MIC, minimum inhibitory concentration.

^*b*^ These data, concerning the proportion of patients with a cefepime concentration exceeding 35 mg/L, relate to the subpopulation of patients under profound sedation and who were therefore clinically unevaluable.

^*c*^ Concerns only patients who underwent an EEG.

^*d*^ Concerns only patients requiring invasive mechanical ventilation (*n *= 158).

### Factors associated with cefepime overexposure

Factors associated with cefepime overexposure in the overall population are presented in Tables S1 and S2.

**Fig 3:**
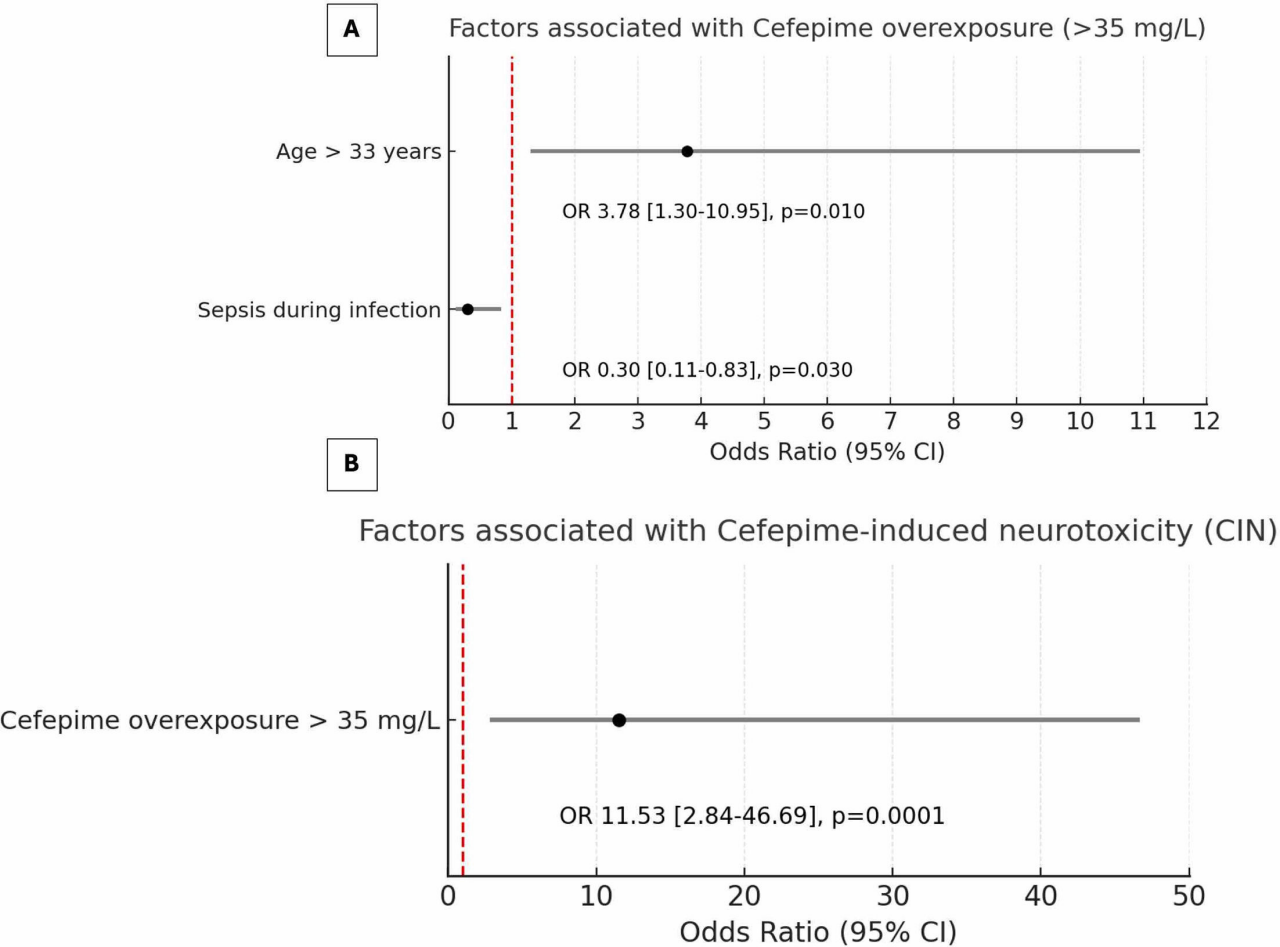
Forest Plot. (**A**) Factors associated with cefepime overexposure in the ARC group. (**B**) Factors associated with cefepime-induced neurotoxicity in the ARC group. OR = odds ratio; [ ] = confidence interval.

Given that a substantial proportion of patients with normal renal clearance (50%) and ARC (32.1%) still experienced cefepime overexposure, we also aimed to identify specific risk factors within these populations (Fig. 3; Table S3; Fig. S2). No risk factors for cefepime overexposure could be identified in patients with normal renal clearance (Table S4).

### Cefepime-induced neurotoxicity: clinical presentation, risk factors, and concentration threshold

Among the 121 (74.7%) clinically evaluable patients, 43 (36.1%) developed a CIN (Fig. S3). The prevalence of CIN varied significantly across the renal clearance groups (*P* = 0.01) (Table 2). Somnolence was the most common manifestation, occurring in 40 patients, among which 22 (55%) had life-threatening coma (Fig. S4). No seizure under cefepime treatment was diagnosed. Among the 27 (16.7%) patients who underwent EEG monitoring, 8 (28.6%) had pathological patterns.

Risk factors for CIN in the overall population are presented in Tables S2 and S5; Fig. S5. Within the ARC group, cefepime overexposure (>35 mg/L) remained the sole predictor of CIN (OR 11.53, 95% CI 2.84–46.69; *P* = 0.0001) (Fig. 3; Table S6). We were not able to identify any risk factors of CIN in the normal renal clearance group (Table S7).

**Fig 4:**
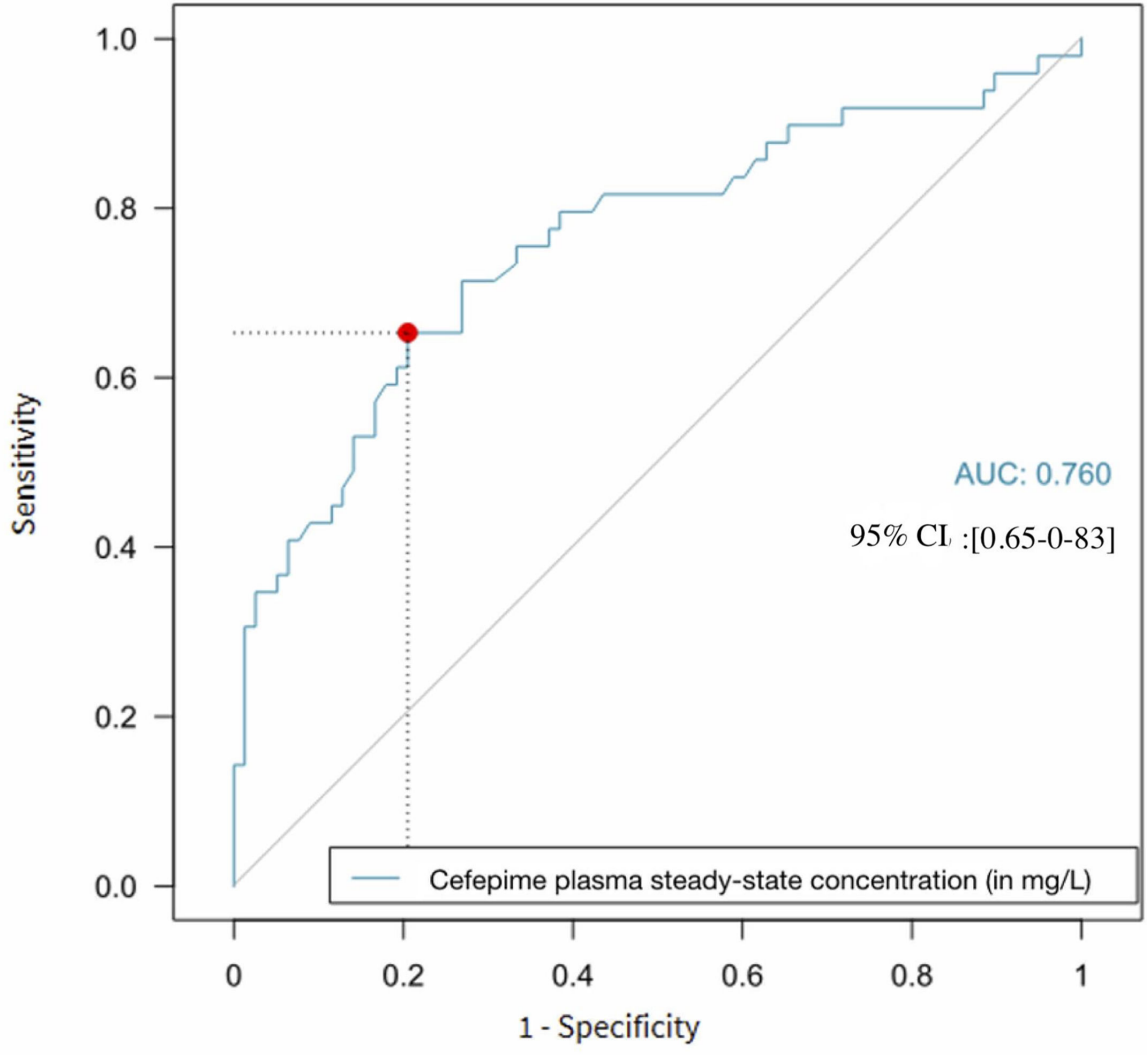
Receiver operating characteristic curve for a plasma cefepime concentration threshold in predicting cefepime-induced neurotoxicity in patients with acute brain injury (n = 130). The x-axis represents 1-specificity, and the y-axis represents sensitivity. The gray diagonal line indicates a random chance prediction. The blue curve illustrates the performance of cefepime plasma steady-state concentration in discriminating CIN. The red dot highlights a potential optimal cutoff point for cefepime plasma steady-state concentration (35 mg/L, corresponding to 1-specificity of 0.23 and sensitivity of 0.66). The AUC is 0.76 (0.65–0.83).

We aimed to determine the specific concentration threshold predictive of CIN in the vulnerable population of patients with acute brain injury (*n* = 130) and designed an ROC curve. The area under the ROC curve was 0.76 (95% CI, 0.65–0.83), and a steady-state concentration threshold of 35 mg/L was identified, yielding a sensitivity of 66% and a specificity of 77% (Fig. 4).

We performed a sensitivity analysis restricted to the 27 patients who underwent EEG monitoring. The ROC analysis in this sub-group yielded an area under the curve of 0.77 (95% CI [0.60–0.95]). The optimal cut-off identified was 36.4 mg/L with a sensitivity of 75% and a specificity of 86% (Fig. S6).

We also conducted an additional exploratory analysis to compare the performance of the 35 mg/L threshold with adjacent cut-off values (Table S8).

### Association between TDM results turnaround time, dosage adjustment, and CIN in the overexposed patients

**Fig 5:**
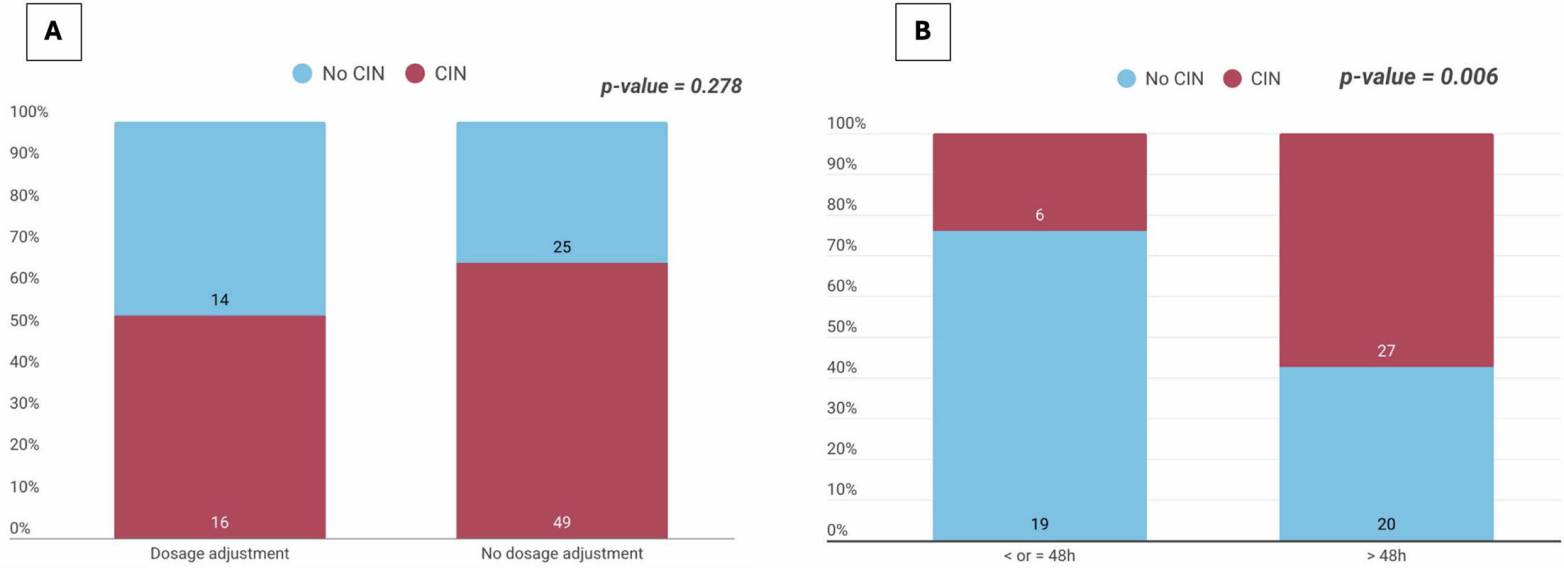
(**A**) Association between cefepime dosage adjustment and the occurrence of cefepime-induced neurotoxicity. The stacked bar chart displays the distribution of cefepime-induced neurotoxicity (CIN, red) and the absence of CIN (No CIN, blue) according to whether a dosage adjustment was performed. The absolute numbers of patients in each subgroup are indicated within the bars. (**B**) Association between therapeutic drug monitoring results turnaround time on the occurrence of cefepime-induced neurotoxicity. This stacked bar chart compares the proportion of patients who developed CIN (red segments) versus those who did not (blue segments), stratified by the time elapsed between cefepime sampling and the availability of the TDM result. Patients were categorized into two groups: time to results ≤48 h (left bar) and time to results >48 h (right bar). The numbers inside the bars indicate the absolute number of patients in each subgroup.

The median TDM result turnaround time was 72 h (48–96). We first analyzed the association between the TDM results turnaround time and the subsequent rate of dosage adjustment in the subpopulation of overexposed patients (*n* = 72). The proportion of patients who received a cefepime dosage adjustment decreased significantly as the time elapsed between cefepime measurement and the availability of the TDM results increased (Fig. S7). We next analyzed the relationship between the decision to perform a dosage adjustment and the occurrence of CIN in the same subpopulation. The incidence of CIN tended to be lower in patients who received a dosage adjustment (24.6% versus 35.9% in patients with no CIN), but this difference did not reach statistical significance (*P* = 0.278) (Fig. 5A).

We investigated the association between TDM results turnaround time and the occurrence of CIN. The incidence of CIN was significantly lower in the group with results obtained within 48 h (24.0%) compared to the group with results obtained after 48 h (57.4%; *P* = 0.006) (Fig. 5B). Finally, the temporal sequence for the 30 patients who underwent dosage adjustment was analyzed to assess the chronological relationship between TDM results and clinical events (symptom onset and dosage adjustment). The temporal sequence is presented in Fig. S8.

### Other secondary outcomes

No significant differences were observed among the groups in terms of ICU mortality, duration of mechanical ventilation, ICU length of stay, treatment failure, or cefepime underexposure (Table 2).

## DISCUSSION

The CEFTOX study provides insights into the clinical consequences of high-dose continuous infusion of cefepime in a population of trauma and brain-injured patients. In this retrospective cohort of critically ill trauma and brain-injured patients, we found that high-dose continuous infusion of cefepime (6 g/day) resulted in plasma concentrations exceeding 35 mg/L in 44.4% of cases. A cefepime steady-state concentration above 35 mg/L was associated with the occurrence of CIN, which was observed in 36.1% of clinically evaluable patients. Despite these findings, no significant differences were observed in terms of ICU mortality, ICU length of stay, or mechanical ventilation duration.

Prior studies have suggested that toxicity is more likely in patients with renal impairment, particularly when trough concentrations exceed 22–38 mg/L during intermittent dosing (10, 31). Unlike intermittent regimens, continuous infusion avoids high peaks but maintains stable plasma concentrations (32). In our cohort, the 35 mg/L threshold was reached in a substantial proportion of patients. Despite continuous infusion, over a third of clinically evaluable patients developed CIN, suggesting that this administration modality does not fully mitigate the risk of neurotoxicity. The concept of a strict “toxic threshold” for cefepime remains debated, as the relationship between plasma concentration and clinical neurotoxicity is complex and influenced by other factors (33). This complexity is further compounded where clinicians aimed for high plasma concentrations to treat infections caused by difficult-to-treat pathogens like *Pseudomonas aeruginosa*. With a breakpoint of 8 mg/L, achieving an optimal PK/PD target would theoretically require plasma concentrations of at least 32 mg/L (4).

This creates a significant clinical dilemma: achieving the necessary concentrations for effective treatment could push the patient into a range associated with CIN. This highlights a need to balance aggressive empirical treatment with the real risk of neurological harm, especially in vulnerable populations. Moreover, our data for patients with a creatinine clearance below 90 mL/min are consistent with the existing literature, suggesting that these patients may benefit from an initial dose reduction (8, 9).

Interestingly, we observed that patients with ARC maintained higher than expected cefepime levels. This contrasts with the common perception that ARC leads to underexposure of beta-lactams. Further PK/PD investigations are warranted to clarify these findings. In our study, all gram-negative bacilli strains were susceptible to cefepime with a MIC of ≤1 mg/L, and only one patient was underdosed with a cefepime concentration of 3.7 (<4× MIC) for a sensitive *Serratia marcescens* VAP successfully treated. Even when considering highest PK/PD targets for gram-negative *Enterobacterales*, 77% of patients with ARC and more than 88% of patients with normal renal clearance would have achieved cefepime steady-state concentrations substantially exceeding the target range. In line with these results, a recent study indicates that a continuous cefepime infusion of 3 g/day offers a favorable benefit-risk profile, even in patients with ARC, by maintaining therapeutic efficacy while reducing the incidence of CIN (34). Besides, another study found no correlation between ARC and cefepime underexposure when administered as a continuous infusion of 6 g/day, unlike other β-lactams such as piperacillin-tazobactam (35). In practice, initial high-dose continuous infusion (6 g/day) appears reasonable for critically ill patients at risk of underexposure, but dose adjustments based on TDM should be rapidly implemented. Thus, our findings, which are consistent with observations from another retrospective cohort, underscore the need for timely TDM results to guide dose adjustment and avoid CIN (25). The issue of delayed assay turnaround times highlights a major limitation in the practical application of TDM for narrow therapeutic index antibiotics like cefepime. Here, the clinical utility of TDM was compromised by the inability to obtain results in an appropriate timing to influence immediate therapeutic decisions.

We sought to identify risk factors for cefepime overexposure specifically within ARC, as this population is theoretically susceptible to underexposure. Age greater than 33 years was identified as a significant risk factor for cefepime overexposure in the ARC group. This cutoff represents the median age of our ARC population, which is particularly young. In the BLING-II sub-study, which aimed to explore the association between ARC and outcomes of patients treated with β-lactams, patients with ARC were also younger, but their median age was 59 years (36). Interestingly, while patients from our cohort were much younger, age was still a critical risk factor for overexposure. In contrast, we identified sepsis as a protective factor against cefepime overexposure in the ARC population. In patients fulfilling sepsis-3 criteria, the profound systemic inflammatory response triggers marked capillary endothelial disruption and vasodilation, resulting in a substantial capillary leak that expands the apparent volume of distribution (Vd) of hydrophilic agents such as cefepime (37). This increased Vd dilutes both peak and trough plasma concentrations, effectively buffering against excessively high cefepime levels. Pharmacokinetic studies have demonstrated that, in septic patients, the Vd of antibiotics can increase by 30% to 50% due to capillary leak (38–40). While sepsis alone can produce capillary leak that dilutes cefepime and protects against transient overexposure despite ARC, septic shock may reverse this protection by impairing renal and nonrenal clearance and creating heterogeneous perfusion that traps the drug intravascularly (41). These pathophysiological shifts lead to drug accumulation and can explain why septic shock, unlike sepsis, does not emerge as a protective factor against cefepime overexposure.

The diagnosis of CIN remains a challenge in the ICU, as its clinical manifestations, particularly encephalopathy, are frequently multifactorial (42).

Our results, showing a 36.1% rate of CIN in the clinically evaluable population (*n* = 121), warrant cautious interpretation. While the scientific community lacks a universal consensus on the definition of CIN, the EEG is often considered necessary for CIN diagnosis (43). The fact that EEG was not systematically performed in our center (only *n* = 27 patients were monitored) constitutes a significant limitation, as it was in some previous studies (26–28).

To counterbalance this limitation and maximize clinical validity, we employed an adapted definition, involving independent evaluation by two neuro-intensivists blinded to TDM results, the strict application of the WHO-UMC causality criteria (“certain” or “probable/likely”), and the systematic exclusion of alternative etiologies (including concurrent sedating agents) (25). Studies that report low rates of CIN, around 2%–6%, cannot be directly compared to our findings (25). First, they combine patients receiving intermittent and continuous infusions (25). Second, their study populations are overrepresented by patients hospitalized in medical wards rather than in ICUs, which may affect the CIN diagnosis (25, 26). Indeed, continuous ICU monitoring and close patient surveillance allow us to identify changes in symptomatology, even if they are mild or moderate. Furthermore, within these same studies, when focusing exclusively on the ICU patient population, the identified rate of CIN was, respectively, 57.1%, 70%, and 59% (25, 26, 33).

### Limitations

To our knowledge, this study represents the largest cohort of ICU patients with acute brain injury treated with cefepime, providing real-world data on cefepime in the ICU. However, some limitations must be acknowledged. First, the retrospective single-center design, focusing on trauma and brain-injured patients, limits the generalizability of the results to other populations. Second, the inter-study variation in CIN definitions, the absence of EEG as a standard of care in our ICU, and the inability to adjust dosage due to delayed TDM results in our center may alter our reported rates of CIN. The temporal sequence observed in the 30 patients with dosage adjustment showed that it was reactive in 14 cases. These reactive changes were driven by the onset of neurotoxicity symptoms as TDM results were reported only after the decision to adjust the dose in most cases. This finding supports the potential value of having rapid TDM turnaround time. Third, our study focused on one cefepime steady-state concentration; while these are commonly used in TDM, they may not fully reflect the overall drug exposure over the dosing interval. Further studies incorporating measures of area under the curve may provide a more comprehensive assessment of the relationship between renal function and cefepime exposure. The choice of the threshold can also be discussed, as several cefepime concentrations are reported in the literature to be associated with CIN (9, 11, 12). The steady-state concentration of 35 mg/L was selected because it was integrated into our local institutional protocol, a decision originally based on both the Huwyler et al. study and the recommendations from the the French Society of Anesthesia and Intensive Care and the French Society of Pharmacology (4, 16). Yet, our ROC analysis in the acute brain injury patients identified 35 mg/L as the optimal predictive threshold for CIN in this group. To address concerns regarding potential circularity in the clinical definition of CIN, due to reliance on symptom reversibility and limited EEG monitoring, we performed a sensitivity analysis restricted to the 27 patients who underwent EEG. This sub-analysis demonstrated that the optimal cutoff was slightly higher, at 36.4 mg/L; nonetheless, this value remains close to the proposed 35 mg/L threshold. In the exploratory analysis, a cefepime concentration of 35 mg/L appeared to offer the best trade-off between sensitivity and specificity. However, given the limited sample size of the EEG-monitored subgroup (*n* = 27) and the exploratory nature of the comparative cutoff analysis, these findings require prospective validation in larger cohorts with systematic EEG monitoring to confirm an optimal threshold in this population.

### Conclusion

High-dose continuous infusion of cefepime leads to steady-state concentration above 35 mg/L, even in patients with ARC. These results highlight the need for early TDM and individualized dose adjustment to balance efficacy and to avoid CIN in the ICU.

## Data Availability

The data sets used and analyzed during the current study are available from the corresponding author on reasonable request.

## SUPPLEMENTAL MATERIAL

Supplemental material aac.01735-25-s0001.docx Supplemental methods; Fig. S1 to S8; Tables S1 to S8.

ASM does not own the copyrights to Supplemental Material that may be linked to, or accessed through, an article. The authors have granted ASM a non-exclusive, world-wide license to publish the Supplemental Material files. Please contact the corresponding author directly for reuse.
