# Supplementary material for: Safety concerns associated with high-dose continuous infusion of cefepime among critically ill patients with mild renal impairment to augmented renal clearance at a level 1 trauma center: CEFTOX study
Source: Antimicrob Agents Chemother. 2026 Feb 12;70(3):e01735-25. doi: 10.1128/aac.01735-25 (PMC12959160; doi:10.1128/aac.01735-25)
Supplement: Supplemental material — Supplemental methods; Fig. S1 to S8; Tables S1 to S8. [file aac.01735-25-s0001.docx]

**Supplementary Material**

Safety concerns associated with high-dose continuous infusion of cefepime among critically ill patients with mild renal impairment to augmented renal clearance at a level 1 trauma center: CEFTOX Study.

Myriam Lamamri (MD, M.Sc.)1 2, Charlotte Rutman (MD, M.Sc.)1, Anais Codorniu (MD; M.Sc.)1, Mathilde Holleville (MD)1, Stéphanie Sigaut (MD, PhD)1 2, Emmanuel Weiss (MD, PhD)1 3, Caroline Jeantrelle (MD)1

**Tables of content**

**Supplementary Methods……………………………………………………………….……2**

**Supplementary AFigure 1…………………………………………………………….….….3**

**Supplementary ATable 1……………………………………………………….……….…..4**

**Supplementary ATable 2………………………………………………………….…….…..5**

**Supplementary ATable 3…………………………………………………………..….…….6**

**Supplementary AFigure 2…………………………………………….………….…...……..7**

**Supplementary ATable 4………………………………………….…………….……….….8**

**Supplementary AFigure 3………………………………………………………………….10**

**Supplementary AFigure 4………………………………………………………………….11**

**Supplementary ATable 5……………………………………………………………..…….12**

**Supplementary AFigure 5…………………………………………………………….…….14**

**Supplementary ATable 6………………………………………………….………….…….15**

**Supplementary ATable 7……………………………………………….…………….…….17**

**Supplementary AFigure 6…………………………………………………………….…….19**

**Supplementary ATable 8……………………………………………….……….…….…….20**

**Supplementary AFigure 7…………………………………………………………….…….21**

**Supplementary AFigure 8…………………………………………………………….…….22**

Supplementary Methods:

Blood samples were collected in lithium heparin tubes, placed on ice and delivered to the toxicology laboratory within 30 minutes. Then, there were centrifuged, mixed 1:1 with a stabilization solution (126mM MOPS, pH 6.8) and stored at −20°C until analysis.

Plasma concentrations of β-lactam antibiotics, including cefepime were quantified using a validated **multi-analyte liquid chromatography–tandem mass spectrometry (LC-MS/MS)** method operated in **multiple-reaction monitoring** mode. Sample preparation involved protein precipitation of 100 µL of human plasma with acetonitrile containing isotopically labeled internal standards for each analyte, followed by centrifugation and dilution of the supernatant with mobile phase. Chromatographic separation was achieved on a reversed-phase C18 column (50 × 2.1 mm, 1.7 µm) maintained at 35 °C using a binary gradient of water and acetonitrile, each containing 0.1 % formic acid. The mass spectrometer was operated in positive electrospray ionization mode, monitoring optimized precursor-to-product ion transitions for each compound.

The lower limits of detection and quantification were 0.2 mg/L and 0.5 mg/L, respectively. The maximal range was 200 mg/L.


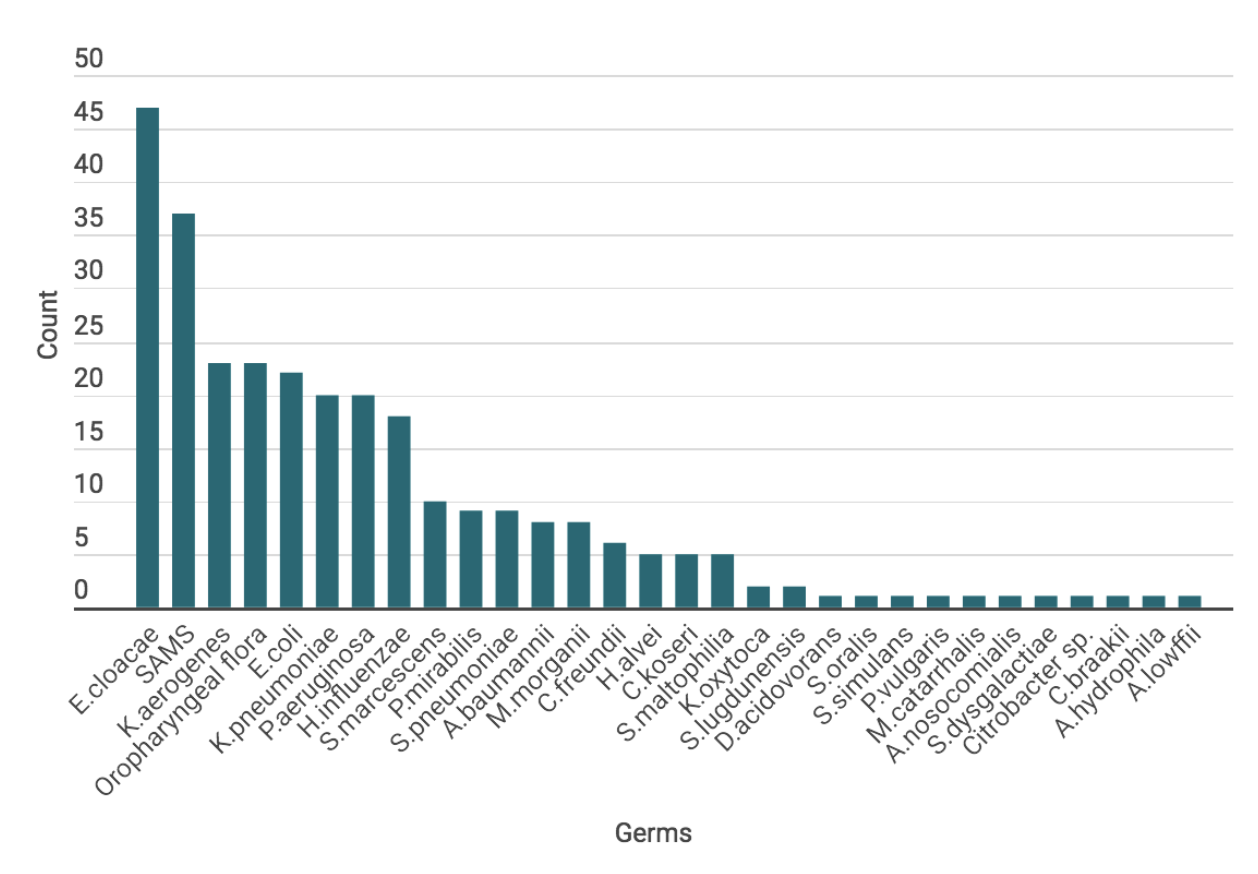


Supplementary AFigure 1 : Distribution of bacterial isolates from different sample sources.

Overall, 162 infectious episodes treated with cefepime were documented, characterized by either monomicrobial or polymicrobial cultures. This bar chart illustrates the frequency of different bacterial species isolated and treated by cefepime. The x-axis represents the identified bacterial germs, while the y-axis indicates the number of times each specific germ was isolated.

Supplementary ATable 1: Univariate analysis of factors associated with cefepime overexposure in the overall population.

|  |  | | **Cefepime overexposure > 35 mg/L** | | | | |
| --- | --- | --- | --- | --- | --- | --- | --- |
|  | Variables | | **Overall** n (%) | | **No** n (%) | **Yes** n (%) | **p-value** |
|  |  |  | **162 (100%)** | | **90 (55.6%)** | **72 (44.4%)** |  |
| Age (years) ; median [IQR] | | | 44 [28-59] | | 38 [23 53] | 47 [35-65] | <0.0001 |
| Male sex ; n (%) | | | 34 (21.0%) | | 20 (22.2%) | 14 (19.4%) | 0.8 |
| BMI (kg/m^2^) ; median [IQR] | | | 25.0 [22.6-29.3] | | 24.7 [22.5-29.3] | 25.3 [22.8-29.3] | 0.1 |
| ASA score ; n (%) | | |  | |  |  | 0.1 |
|  | 1 | | 65 (40.1%) | | 39 (43.3%) | 26 (36.1%) |  |
|  | 2 | | 72 (44.4%) | | 42 (46.7%) | 30 (41.7%) |  |
|  | 3 | | 25 (15.4%) | | 9 (10.0%) | 16 (22.2%) |  |
| SAPS II ; median [IQR] | | | 46 [34-58] | | 45 [34-57] | 47 [31-60] | 0.8 |
| SOFA score ; median [IQR] | | | 6 [4-8] | | 6 [4-8] | 6 [4-9] | 0.7 |
| Reason for ICU admission; n (%) | | |  | |  |  | 0.2 |
|  | Severe Trauma | | 137 (84.6%) | | 78 (86.7%) | 59 (81.9%) |  |
|  | Hemorrhagic stroke | | 13 (8.0%) | | 4 (4.4%) | 9 (12.5%) |  |
|  | Other neurological injuries | | 8 (4.9%) | | 5 (5.6%) | 3 (4.2%) |  |
|  | Sepsis | | 4 (2.5%) | | 3 (3.3%) | 1 (1.4%) |  |
| Traumatic brain injury ; n (%) | | | 109 (67.3%) | | 63 (70.0%) | 46 (63.9%) | 0.5 |
| Hemorrhagic shock at admission ; n (%) | | | 36 (22.2%) | | 21 (23.3%) | 15 (20.8%) | 0.8 |
| Transfusion at admission ; n (%) | | | 43 (26.5%) | | 26 (28.9%) | 17 (23.6%) | 0.6 |
| Site of infection ; n (%) | | |  |  | |  | 0.4 |
|  | VAP | | 127 (79.4%) | 73 (81.1%) | | 54 (75.0%) |  |
|  | Limb surgical site infection | | 13 (8.0%) | 7 (7.8%) | | 6 (8.3%) |  |
|  | Spine surgical site infection | | 9 (5.6%) | 4 (4.4%) | | 5 (6.9%) |  |
|  | Meningitis | | 3 (1.8%) | 2 (2.2%) | | 1 (1.4%) |  |
|  | Bacteriemia | | 3 (1.8%) | 0 (0.0%) | | 3 (4.2%) |  |
|  | Peritonitis | | 3 (1.8%) | 2 (2.2%) | | 1 (1.4%) |  |
|  | Cerebral empyema | | 2 (1.2%) | 0 (0%) | | 2 (2.8%) |  |
|  | Necrotizing fasciitis | | 1 (0.6%) | 1 (1.1%) | | 0 (0.0%) |  |
|  | Prostatitis | | 1 (0.6%) | 1 (1.1%) | | 0 (0.0%) |  |
| Septic shock during the infection ; n (%) | | | 38 (23.5%) | 18 (20.0%) | | 20 (27.8%) | 0.3 |
| Sepsis during the infection; n (%) | | | 96 (59.3%) | 58 (64.4%) | | 38 (52.8%) | 0.2 |
| Fluid balance assessment at the initiation of cefepime (mL) ; median [IQR] | | | -275 [-1200- 350] | -350 [-1195- 425] | | -100 [-1200-340] | 0.9 |
| Maximum infusion rate of norepinephrine (in γ/kg/min) ; median [IQR] | | | 0.41 [0.22-0.79] | 0.46 [0.29-0.76] | | 0.4 [0.2- 0.81] | 0.9 |
| Albuminemia at cefepime initiation (g/L) ; median [IQR] | | | 22.4 [19.3-26.9] | 22.4 [19.3-26.2] | | 22.3 [19.5-  27.9] | 0.4 |
| Hematocrit at cefepime initiation (%) ; median [IQR] | | | 25.7 [23.3-29.6] | 25.0 [23.0-28.3] | | 26.5 [24.1-30.8] | 0.02 |
| Protidemia at cefepime initiation (g/L) ; median [IQR] | | | 62 [56-69] | 62 [56-69] | | 62 [55-70] | 0.8 |
| Creatinine clearance concomitant with cefepime TDM (mL/min) ; median [IQR] | | | 154 [121-198.5] | 164 [131.5-213.7] | | 129 [86- 168] | <0.0001 |
| Plasma creatinine at admission (μmol/L) ; median [IQR] | | | 78 [60-99.8] | 51 [39.2-59.7] | | 59 [44-79] | <0.0001 |
| Classification of creatinine clearance; n (%) | | |  |  | |  | <0.0001 |
|  | | >150 ml/min | 84 (49.1%) | 57 (63.3%) | | 27 (33.3%) |  |
|  | | 90-150 ml/min | 62 (36.3%) | 31 (34.4%) | | 31 (38.3%) |  |
|  | | 60-90 ml/min | 16 (9.9%) | 2 (12.5%) | | 14 (87.5%) |  |

N = number ; IQR = interquartile range ; BMI = Body Mass Index ; ASA = American Society of Anesthesiologists ; ICU = Intensive Care Unit ; SAPS II = Simplified Acute Physiology Score II ; SOFA = Sequential Organ Failure Assessment ; VAP = ventilator associated pneumonia ; TDM = Therapeutic Drug Monitoring.

Supplementary ATable 2: Table 2: Multivariate analyses of factors associated with cefepime overexposure (>35 mg/L) and cefepime-induced neurotoxicity in the overall population.

| **Factors associated** | **Odds ratio** | **95% CI** | **p-value** |
| --- | --- | --- | --- |
| **Factors associated with cefepime overexposure (> 35 mg/L) in the overall population** |  |  |  |
| Augmented renal clearance (> 150 ml/min) | 0.47 | [0.24-0.93] | 0.03 |
| Mild renal impairment (60-90 ml/min) | 7.00 | [1.47-33.41] | 0.01 |
| **Factors associated with CIN in the overall population** |  |  |  |
| Cefepime overexposure > 35 mg/L | 5.76 | [2.42-13.72] | 0.0001 |
| Age > 33 years | 2.54 | [1.03-6.25] | 0.01 |
| Creatinine clearance > 138 ml/min | 0.78 | [0.31-1.92] | 0.59 |

CI = confidence interval; CIN = cefepime-induced neurotoxicity.

Supplementary ATable 3 : Univariate analysis of factors associated with cefepime overexposure in the ARC group.

|  |  | **Cefepime overexposure > 35 mg/L** | | | |
| --- | --- | --- | --- | --- | --- |
|  | Variables | **Overall n**(%) | **No n**(%) | **Yes n**(%) | **p-value** |
|  |  | **84 (100%)** | **57 (68%)** | **27 (32%)** |  |
| Age (years) ; median [IQR] | | 36 [23-47] | 31 [21-47] | 42 [30-52] | 0.08 |
| Male sex ; n(%) | | 14 (17%) | 10 (18%) | 4 (15%) | 1 |
| BMI (kg/m^2^) ; median [IQR] | | 25 [23-29] | 25 [22-29] | 25 [23-29] | 0.5 |
| ASA score ; n(%) | |  |  |  | 0.07 |
|  | 1 | 41 (49%) | 29 (51%) | 12 (44%) |  |
|  | 2 | 38 (45%) | 27 (47%) | 11 (41%) |  |
|  | 3 | 5 (6%) | 1 (2%) | 4 (15%) |  |
| SAPS II ; median [IQR] | | 45 [34-58] | 45 [34-60] | 42 [32-52] | 0.4 |
| Reason for ICU admission ; n(%) | |  |  |  | 0.8 |
|  | Severe trauma | 76 (90%) | 52 (91%) | 24 (89%) |  |
|  | Hemorrhagic stroke | 3 (4%) | 2 (4%) | 1 (4%) |  |
|  | Other neurological injuries | 5 (6%) | 3 (5%) | 2 (7%) |  |
|  | Sepsis | 0 (0%) | 0 (0%) | 0 (0%) |  |
| Traumatic brain injury ; n(%) | | 65 (77%) | 44 (77%) | 21 (78%) | 1 |
| Hemorrhagic shock at admission ; n(%) | | 13 (15%) | 10 (18%) | 3 (11%) | 0.5 |
| Plasma creatinine at admission (μmol/L) ; median [IQR] | | 74 [58-96] | 76 [64-103] | 66 [56-86] | 0.2 |
| Transfusion ; n(%) | | 18 (21%) | 13 (23%) | 5 (19%) | 0.8 |
| Site of infection ; n(%) | |  |  |  | 0.3 |
|  | VAP | 67 (80%) | 47 (82%) | 20 (74%) |  |
|  | Limb surgical site infection | 5 (6%) | 4 (7%) | 1 (4%) |  |
|  | Spine surgical site infection | 4 (5%) | 2 (4%) | 2 (7%) |  |
|  | Meningitis | 3 (4%) | 2 (4%) | 1 (4%) |  |
|  | Bacteriemia | 1 (1%) | 0 (0%) | 1 (4%) |  |
|  | Peritonitis | 2 (2%) | 0 (0%) | 2 (7%) |  |
|  | Cerebral empyema | 1 (1%) | 1 (2%) | 0 (0%) |  |
|  | Necrotizing fasciitis | 1 (1%) | 1 (2%) | 0 (0%) |  |
| Septic shock during the infection ; n(%) | | 14 (17%) | 7 (12%) | 7 (26%) | 0.1 |
| Sepsis during the infection ; n(%) | | 51 (61%) | 39 (68%) | 12 (44%) | 0.05 |
| SOFA score ; median [IQR] | | 6 [4-7] | 6 [4-7] | 6 [4-8] | 0.6 |
| Albuminemia at cefepime initiation (g/L) ; median [IQR] | | 23 [20-28] | 23 [20-27] | 23 [21-28] | 0.5 |
| Protidemia at cefepime initiation (g/L) ; median [IQR] | | 63 [56-69] | 63 [56-66] | 66 [57-70] | 0.3 |
| Fluid balance assessment at the initiation of cefepime (mL) ; median [IQR] | | -572.5 [-1400-340] | -550 [-1225- 62.5] | -672.5 [-1475-462.5] | 0.1 |
| Maximum infusion rate of norepinephrine (in γ/kg/min) ; median [IQR] | | 0.41 [0.24-0.67] | 0.31 [0.26-0.54] | 0.43 [0.3-0.92] | 0.4 |

N = number ; IQR = interquartile range ; BMI = Body Mass Index ; ASA = American Society of Anesthesiologists ; ICU = Intensive Care Unit ; SAPS II = Simplified Acute Physiology Score II ; SOFA = Sequential Organ Failure Assessment ; VAP = ventilator associated pneumonia.

Supplementary AFigure 2 : Receiver Operating Characteristic (ROC) Curve for age in predicting cefepime overexposure in the ARC group.


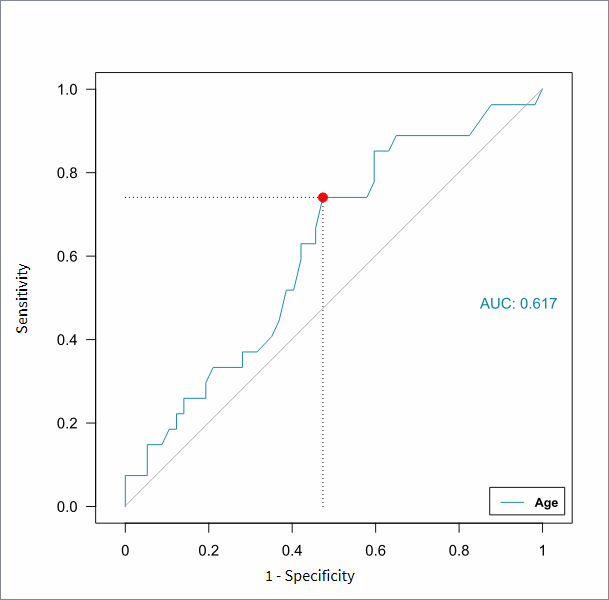


This figure displays ROC curve for age as a predictor of cefepime overexposure. The x-axis represents 1 - specificity, and the y-axis represents sensitivity. The grey diagonal line indicates a random chance prediction. The blue curve illustrates the performance of age in discriminating cefepime overexposure. The red dot highlights an optimal cutoff point for age (33 years, corresponding to 1 - specificity of 0.47 and sensitivity of 0.74). The area under the curve (AUC) is 0.62, suggesting a fair discriminatory ability.

Supplementary ATable 4 : Univariate analysis of factors associated with cefepime overexposure in the normal renal clearance group.

|  |  | **Cefepime overexposure > 35 mg/L** | | | |
| --- | --- | --- | --- | --- | --- |
|  |  | **Overall** ; n(%) | **No** ; n(%) | **Yes** ; n(%) | **p-value** |
| Variables |  | **62 (100%)** | **31 (50.0%)** | **31 (50.0%)** |  |
| Age (years) median [IQR] |  | 47 [31-59] | 50 [31-56] | 47 [33-62] | 0.6 |
| Male sex ; n(%) | | 16 (25.8%) | 9 (29.0%) | 7 (22.6%) | 0.8 |
| BMI (kg/m^2^) ; median [IQR] |  | 24.5 [22.2-27.6] | 24.2 [22.1- 29.3] | 24.7 [22.4-26.6] | 0.7 |
| ASA score ; n(%) |  |  |  |  | 0.9 |
|  | 1 | 22 (35.5%) | 10 (32.3%) | 12 (38.7%) |  |
|  | 2 | 26 (41.9%) | 14 (45.2%) | 12 (38.7%) |  |
|  | 3 | 14 (22.6%) | 7 (22.6%) | 7 (22.6%) |  |
| SAPS II ; median [IQR] |  | 45 [33-54] | 45 [38-51] | 47 [26-62] | 0.7 |
| SOFA score ; median [IQR] |  | 6 [4-8] | 7 [4-9] | 6 [4-8] | 0.5 |
| Reason for ICU admission ; n(%) | | |  |  | 0.6 |
|  | Severe trauma | 51 (82.3%) | 25 (80.6%) | 26 (83.9%) |  |
|  | Hemorrhagic stroke | 6 (9.7%) | 2 (6.5%) | 4 (12.9%) |  |
|  | Other neurological injuries | 3 (4.8%) | 2 (6.5%) | 1 (3.2%) |  |
|  | Sepsis | 2 (3.2%) | 2 (6.5%) | 0 (0%) |  |
| Traumatic brain injury ; n(%) | | 38 (61.3%) | 18 (58.1%) | 20 (64.5%) | 0.8 |
| Hemorrhagic shock at admission ; n(%) |  | 19 (30.6%) | 11 (35.5%) | 8 (25.8%) | 0.6 |
| Site of infection ; n(%) | |  |  |  | 0.3 |
|  | VAP | 49 (79.0%) | 25 (80.6%) | 24 (77.4%) |  |
|  | Limb surgical site infection | 6 (9.7%) | 3 (9.7%) | 3 (9.7%) |  |
|  | Spine surgical site infection | 2 (3.2%) | 1 (3.2%) | 1 (3.2%) |  |
|  | Bacteriemia | 3 (4.8%) | 0 (0%) | 3 (9.7%) |  |
|  | Peritonitis | 2 (3.2%) | 2 (6.5%) | 0 (0%) |  |
| Septic shock during the infection ; n(%) |  | 17 (27.4%) | 9 (29.0%) | 8 (25.8%) | 1 |
| Sepsis during the infection ; n(%) |  | 38 (61.3%) | 19 (61.3%) | 19 (61.3%) | 1 |
| Protidemia at cefepime initiation (g/L) ; median [IQR] |  | 62 [55-71] | 62 [55-71] | 62 [56-71] | 0.8 |
| Hematocrit at cefepime initiation (%) ; median [IQR] |  | 26 [23-30] | 26 [23-27] | 27 [24-30] | 0.1 |
| Albuminemia at cefepime initiation (g/L) ; median [IQR] | | 22 [19-26] | 21 [18-25] | 23 [19-29] | 0.2 |
| Fluid balance assessment at the initiation of cefepime (mL) ; median [IQR] | | -100 [-1025-425] | -90 [-945-400] | -110 [-1075-510] | 0.9 |
| Plasma creatinine at admission (μmol/L) ; median [IQR] | | 79 [63-99.8] | 81 [75-117] | 80 [77.5-108] | 0.6 |

N = number ; IQR = interquartile range ; BMI = Body Mass Index ; ASA = American Society of Anesthesiologists ; ICU = Intensive care unit ; SAPS II = Simplified Acute Physiology Score II ; SOFA = Sequential Organ Failure Assessment ; VAP = ventilator associated pneumonia.

Supplementary AFigure 3: Distribution of adverse neurological events classified as related to CIN by causality assessment (WHO Criteria).


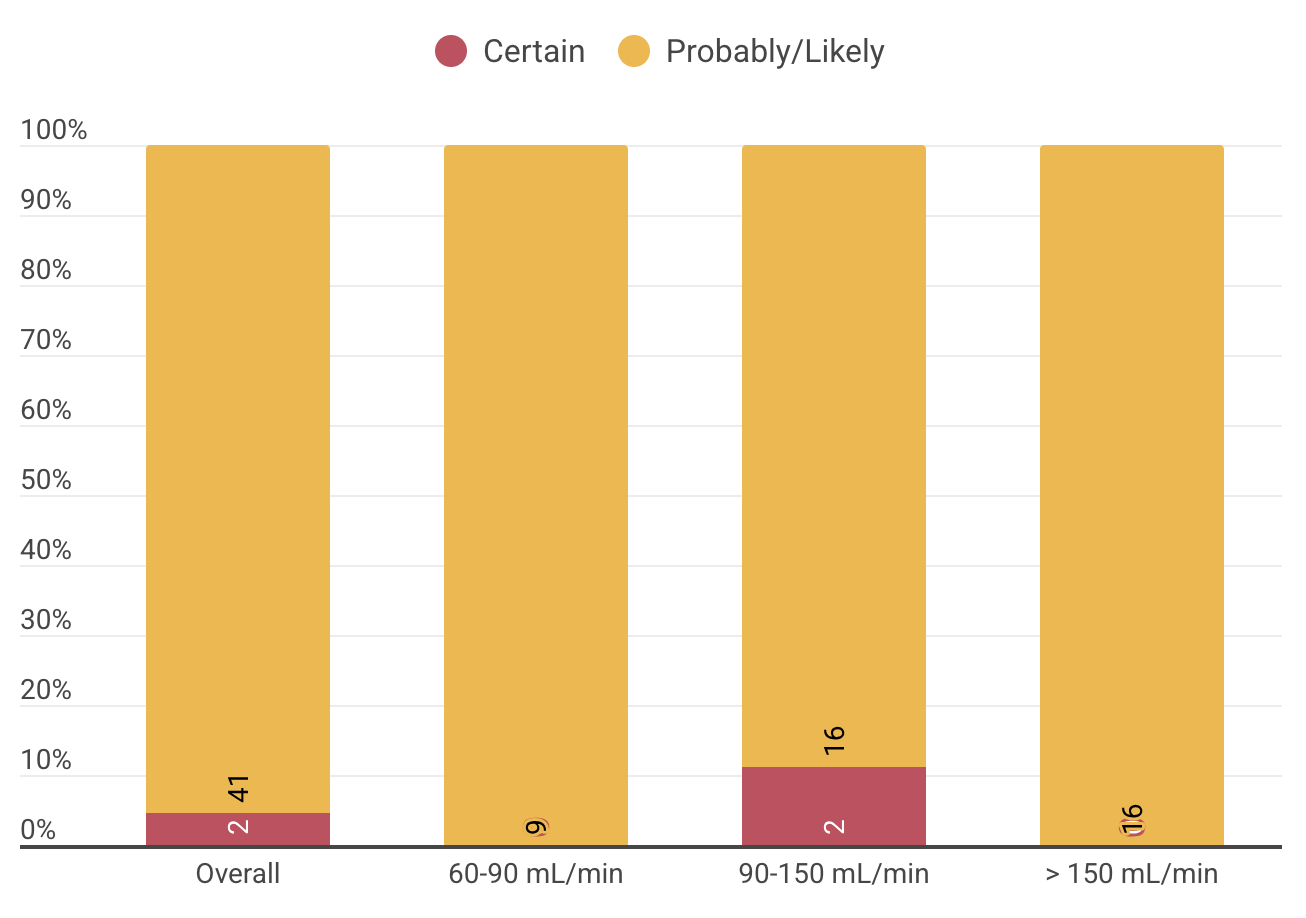


We defined, using World Health Organization (WHO) causality assessment, neurological symptoms as 'certain' and 'probably/likely' CIN-related events.

The graph presents the proportion of patients with neurological events classified as 'Certain' (Red) or 'Probable/Likely' (Yellow) according to the World Health Organization (WHO) causality assessment. The data is stratified by baseline CrCl groups. The numbers shown inside the bars represent the absolute patient count within each CrCl category.

Supplementary AFigure 4: Clinical manifestations of cefepime-induced neurotoxicity by severity grade according to common terminology criteria for adverse events (CTCAE).


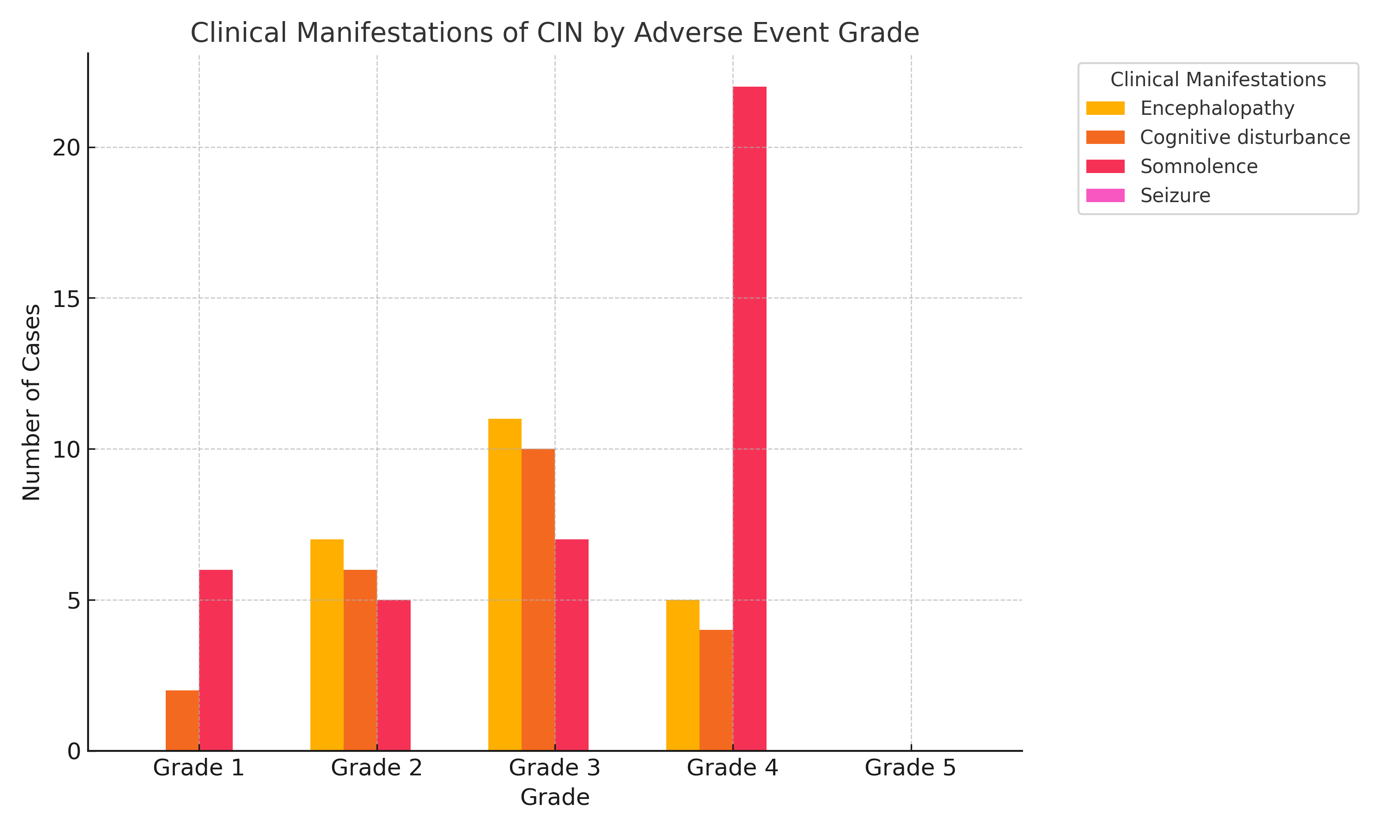


This bar chart presents the number of patients experiencing each specific clinical manifestation of CIN, categorized by severity grade (Grade 1 to 5) as defined by the CTCAE (25).

Supplementary ATable 5: Univariate analysis of factors associated with cefepime-induced neurotoxicity in the overall population.

|  |  | **Cefepime-induced neurotoxicity** | | | |
| --- | --- | --- | --- | --- | --- |
|  | Variables | **Overall** n(%) | **No** n(%) | **Yes** n(%) | **p-value** |
|  |  | **127 (100%)** | **84 (66.1%)** | **43 (33.8%)** |  |
| Age (years) ; median [IQR] | | 44 [28-60] | 34 [23-52] | 52 [37-64] | <0.0001 |
| Male sex ; n(%) | | 29 (22.8%) | 16 (20.5%) | 13 (26.5%) | 0.5 |
| BMI (kg/m^2^) ; median [IQR] | | 25 [22-29] | 25 [21-29] | 26 [23-31] | 0.2 |
| ASA score ; n(%) | |  |  |  | 0.05 |
|  | 1 | 48 (37.8%) | 35 (44.9%) | 13 (26.5%) |  |
|  | 2 | 56 (44.1%) | 33 (42.3%) | 23 (46.9%) |  |
|  | 3 | 23 (18.1%) | 10 (12.8%) | 13 (26.5%) |  |
| SAPS II ; median [IQR] | | 46 [33 - 60] | 42 [32 - 54] | 53 [38 - 64] | 0.01 |
| Reason for ICU admission ; n(%) | |  |  |  | 0.4 |
|  | Severe trauma | 90 (70.9%) | 59 (75.6%) | 31 (63.3%) |  |
|  | Hemorrhagic stroke | 13 (10.2%) | 6 (7.7%) | 7 (14.3%) |  |
|  | Other neurological injuries | 19 (15.1%) | 11 (14.2%) | 4 (16.3%) |  |
|  | Sepsis | 4 (2.4%) | 2 (2.6%) | 2 (4.1%) |  |
| Traumatic brain injury ; n(%) | | 78 (61.4%) | 48 (61.5%) | 30 (61.2%) | 1 |
| Hemorrhagic shock at admission ; n(%) | | 28 (22.0%) | 18 (23.1%) | 10 (20.4%) | 0.8 |
| Transfusion ; n(%) | | 33 (26.0%) | 21 (26.9%) | 12 (24.5%) | 0.8 |
| Plasma creatinine at admission (μmol/L) ; median [IQR] | | 78 [60-102] | 77 [59.2-102.2] | 80 [61-102] | 0.7 |
| Fibrinogen (g/L) ; n(%) | | 19 (15.0%) | 16 (20.5%) | 3 (6.1%) | 0.04 |
| Site of infection ; n(%) | |  |  |  | 0.3 |
|  | VAP | 91 (71.7%) | 58 (74.4%) | 33 (67.3%) |  |
|  | Limb surgical site infection | 14 (11.0%) | 9 (11.5%) | 5 (10.2%) |  |
|  | Spine surgical site infection | 9 (7.1%) | 7 (9.0%) | 2 (4.1%) |  |
|  | Meningitis | 3 (2.4%) | 1 (1.3%) | 2 (4.1%) |  |
|  | Bacteriemia | 4 (3.1%) | 1 (1.3%) | 3 (6.1%) |  |
|  | Peritonitis | 3 (2.4%) | 1 (1.3%) | 2 (4.1%) |  |
|  | Cerebral empyema | 1 (0.8%) | 0 (0%) | 1 (2.0%) |  |
|  | Necrotizing fasciitis | 1 (0.8%) | 0 (0%) | 1 (2.0%) |  |
| Cefepime overexposure > 35 mg/L; n(%) | | 65 (51.2%) | 26 (33.3%) | 39 (79.6%) | <0.0001 |
| Cefepime plasma concentration (mg/L) ; median [IQR] | | 35 [24.6-47.6] | 30.6 [21.6-38.1] | 45.4 [35.6-65.4] | <0.0001 |
| Duration of cefepime treatment (days) ; median [IQR] | | 7 [4-7] | 7 [3.5-7] | 7 [5-7] | 1 |
| Septic shock during the infection ; n(%) | | 25 (19.7%) | 13 (16.7%) | 12 (24.5%) | 0.4 |
| Sepsis during the infection ; n(%) | | 73 (57.5%) | 44 (56.4%) | 29 (59.2%) | 0.8 |
| SOFA score ; median [IQR] | | 5 [4-7] | 5 [4-7] | 6 [4-9] | 0.4 |
| Fluid balance assessment at the initiation of cefepime ; median [IQR] | | -200 [-1000-462.5] | -201 [-1142.5-337.5] | -50 [-900-625] | 0.3 |
| Maximum infusion rate of norepinephrine (in γ/kg/min) ; median [IQR] | | 0.4 [0.2-0.7] | 0.4 [0.3-0.5] | 0.2 [0.2-0.8] | 0.6 |
| Creatinine clearance concomitant with cefepime TDM (ml/min) ; median [IQR] | | 139 [114-189.5] | 157.5 [125.8-203] | 122 [86-158] | <0.0001 |
| ARC ; n(%) | | 57 (44.9%) | 41 (52.6%) | 16 (32.7%) | 0.04 |
| Normal renal clearance ; n(%) | | 49 (38.6%) | 31 (39.7%) | 18 (36.7%) | 0.8 |
| Mild renal impairment ; n(%) | | 9 (11.4%) | 1 (0.8%) | 8 (3.9%) | 0.2 |
| Albuminemia at cefepime initiation (g/L) ; median [IQR] | | 22 [19-27] | 22 [19-27] | 23 [21- 28] | 0.4 |
| Protidemia at cefepime initiation(g/L) ; median [IQR] | | 62 [56-69] | 62 [56-69] | 61 [52-70] | 0.3 |
| Hematocrit at cefepime initiation (%) ; median [IQR] | | 26 [23-30] | 26 [24-30] | 25 [23-31] | 0.2 |

N = number; IQR = interquartile range ; BMI = Body Mass Index ; ASA = American Society of Anesthesiologists ; SAPS II = Simplified Acute Physiology Score II ; SOFA = Sequential Organ Failure Assessment ; VAP = ventilator associated pneumonia ; TDM = Therapeutic Drug Monitoring ; ARC = augmented renal clearance.

Supplementary AFigure 5: Receiver Operating Characteristic (ROC) curve for age in predicting cefepime-induced neurotoxicity in the overall population.


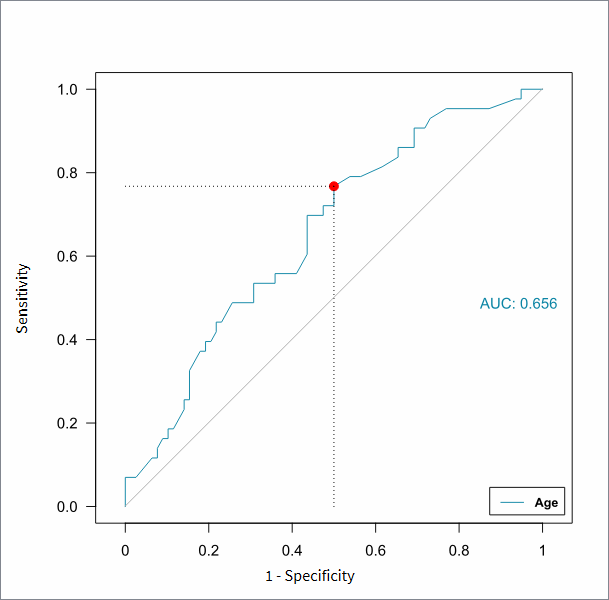


This figure displays ROC curve for age as a predictor of CIN. The x-axis represents 1 - specificity, and the y-axis represents sensitivity. The grey diagonal line indicates a random chance prediction. The blue curve illustrates the performance of age in discriminating CIN. The red dot highlights an optimal cutoff point for age (33 years, corresponding to 1 - specificity of 0.50 and sensitivity of 0.77). The AUC is 0.65, suggesting a fair discriminatory ability.

Supplementary ATable 6: Univariate analysis of factors associated with cefepime-induced neurotoxicity in augmented renal clearance group.

|  |  | | **Cefepime-induced neurotoxicity** | | | | | | |
| --- | --- | --- | --- | --- | --- | --- | --- | --- | --- |
|  | Variables | | **Overall** n(%) | **No** n(%) | | | **Yes** n(%) | | **p-value** |
|  |  |  | **57 (100%)** | **31 (71.9%)** | | | **16 (28.1%)** | |  |
| Age (years) ; median [IQR] | | | 33 [23-45] | 31 [21-45] | | | 39 [28-44] | | 0.1 |
| Male sex ; n(%) | | | 11 (19.3%) | 7 (17.1%) | | | 4 (25.0%) | | 0.5 |
| BMI (kg/m^2^) ; median [IQR] | | | 24.8 [22.6-29.3] | 25 [22-27] | | | 26.2 [23-28.7] | | 0.6 |
| ASA score ; n(%) | | | 1 [1-2] | 1 [1-2] | | | 2 [1-2] | | 0.5 |
| SAPS II ; median [IQR] | | | 42 [33-57] | 42 [33-54] | | | 46 [34-62] | | 0.5 |
| SOFA score ; median [IQR] | | | 5 [4-6] | 5 [4-6] | | | 5 [3-6] | | 0.8 |
| Reason for ICU admission ; n(%) | | |  |  | | |  | | 1 |
|  | Severe trauma | | 50 (87.7%) | 36 (81.9%) | | | 14 (87.5%) | |  |
|  | Hemorrhagic stroke | | 3 (5.3%) | 2 (4.9%) | | | 1 (6.2%) | |  |
|  | Other neurological injuries | | 2 (3.5%) | 3 (7.3%) | | | 1 (6.2%) | |  |
|  | Sepsis | | 0 (0%) | 0 (0%) | | | 0 (0%) | |  |
| Traumatic brain injury ; n(%) | | | 43 (75.4%) | 30 (73.2%) | | | 13 (81.2%) | | 0.7 |
| Hemorrhagic shock at admission ; n(%) | | | 9 (15.8%) | 7 (17.1%) | | | 2 (12.5%) | | 1 |
| Transfusion ; n(%) | | | 11 (19.3%) | 9 (21.9%) | | | 2 (12.5%) | | 0.7 |
| Cefepime plasma concentration (mg/L) ; median [IQR] | | | 28.8 [20-36.8] | 27.6 [19-32.2] | | | 42.3 [25.9-46.2] | | <0.0001 |
| Cefepime overexposure > 35 mg/L ; n(%) | | | 18 (31.6%) | 7 (17.1%) | | | 11 (68.7%) | | <0.0001 |
| Septic shock during the infection ; n(%) | | | 4 (7.0%) | 3 (7.3%) | | | 1 (6.2%) | | 1 |
| Sepsis during the infection ; n(%) | | | 34 (59.6%) | 24 (58.5%) | | | 10 (62.5%) | | 1 |
| Site of infection ; n(%) | | |  |  | | |  | | 0.3 |
|  | VAP | 43 (75.4%) | | | 31 (75.6%) | 12 (75.0%) | |  | |
|  | Limb surgical site infection | 4 (7.0%) | | | 4 (9.8%) | 0 (0%) | |  | |
|  | Spine surgical site infection | 4 (7.0%) | | | 3 (7.3%) | 1 (6.2%) | |  | |
|  | Meningitis | 1 (1.7%) | | | 1 (2.4%) | 0 (0%) | |  | |
|  | Bacteriemia | 2 (3.5%) | | | 1 (2.4%) | 1 (6.2%) | |  | |
|  | Peritonitis | 1 (1.7%) | | | 0 (0%) | 1 (6.2%) | |  | |
|  | Cerebral empyema | 1 (1.7%) | | | 0 (0%) | 1 (6.2%) | |  | |
|  | Necrotizing fasciitis | 1 (1.7%) | | | 1 (2.4%) | 0 (0%) | |  | |
| Plasma creatinine at admission (μmol/L) ; median [IQR] | | 46 [38-57] | | | 45 [38-59] | 47 [39-51.3] | | 0.5 | |
| Duration of cefepime treatment (days) ; median [IQR] | | 7 [4-7] | | | 7 [3-7] | 7 [6-7] | | 0.3 | |
| Creatinine clearance concomitant with cefepime TDM (mL/min) ; median [IQR] | | 197 [165- 224] | | | 197 [175-227] | 185.5 [158-210.2] | | 0.2 | |

N = number ; IQR = interquartile range ; BMI = Body Mass Index ; ASA = American Society of Anesthesiologists ; SAPS II = Simplified Acute Physiology Score II ; SOFA = Sequential Organ Failure Assessment ; VAP = ventilator associated pneumonia ; TDM = Therapeutic Drug Monitoring ; ARC = augmented renal clearance.

Supplementary ATable 7: Univariate analysis of factors associated with cefepime-induced neurotoxicity in normal renal clearance group.

|  |  | | **Cefepime-induced neurotoxicity** | | | | | |
| --- | --- | --- | --- | --- | --- | --- | --- | --- |
|  |  | | **Overall** | **No** | | | **Yes** | **p-value** |
|  |  |  | **49 (100%)** | **31 (63.3%)** | | | **16 (36.7%)** |  |
| Age (years) ; median [IQR] | | | 45 [30-59] | 44 [25-53] | | | 50 [37-60] | 0.3 |
| Male sex ; n(%) | | | 11 (22.4%) | 7 (22.6%) | | | 4 (22.2%) | 1 |
| BMI (kg/m^2^) ; median [IQR] | | | 24 [22-27] | 24 [20-27] | | | 25 [23-28] | 0.3 |
| ASA score ; n(%) | | |  |  | | |  | 0.6 |
| 1 | | | 18 (36.7%) | 13 (41.9%) | | | 5 (27.8%) |  |
| 2 | | | 20 (40.8%) | 12 (38.7%) | | | 8 (44.4%) |  |
| 3 | | | 11 (22.4%) | 6 (19.3%) | | | 5 (27.8%) |  |
| SAPS II ; median [IQR] | | | 46 [37-57] | 43 [31-53] | | | 50 [39-62] | 0.2 |
| SOFA score ; median [IQR] | | | 6 [4-8] | 5 [4-7] | | | 6 [5-8] | 0.2 |
| Reason for ICU admission ; n(%) | | |  |  | | |  | 0.6 |
|  | Severe trauma | | 40 (81.6%) | 27 (87.1%) | | | 13 (72.2%) |  |
|  | Hemorrhagic stroke | | 5 (10.2%) | 2 (6.4%) | | | 3 (16.7%) |  |
|  | Other neurological injuries | | 2 (4.1%) | 1 (3.2%) | | | 1 (5.6%) |  |
|  | Sepsis | | 2 (4.1%) | 1 (3.2%) | | | 1 (5.6%) |  |
| Traumatic brain injury ; n(%) | | | 29 (59.2%) | 17 (54.8%) | | | 12 (66.7%) | 0.5 |
| Hemorrhagic shock at admission ; n(%) | | | 15 (30.6%) | 11 (35.5%) | | | 4 (22.2%) | 0.5 |
| Cefepime overexposure > 35 mg/L ; n(%) | | | 28 (57.1%) | 15 (48.4%) | | | 13 (72.2%) | 0.1 |
| Septic shock during the infection ; n(%) | | | 12 (24.5%) | 7 (22.6%) | | | 5 (27.8%) | 0.7 |
| Sepsis during the infection ; n(%) | | | 30 (61.2%) | 17 (54.8%) | | | 13 (72.2%) | 0.4 |
| Site of infection ; n(%) | | |  |  | | |  | 0.06 |
|  | VAP | 36 (73.5%) | | | 23 (74.19%) | 13 (72.2%) | |  |
|  | Limb surgical site infection | 6 (12.2%) | | | 5 (16.1%) | 1 (5.6%) | |  |
|  | Spine surgical site infection | 2 (4.1%) | | | 2 (6.4%) | 0 (0%) | |  |
|  | Meningitis | 3 (6.1%) | | | 0 (0%) | 3 (16.7%) | |  |
|  | Bacteriemia | 2 (4.1%) | | | 1 (3.2%) | 1 (5.6%) | |  |
|  | Peritonitis | 35 (71.4%) | | | 23 (74.2%) | 12 (66.7%) | |  |
| Plasma creatinine at admission (μmol/L) ; median [IQR] | | 51 [44-68] | | | 50 [43-66] | 64 [46.1-80.7] | | 0.09 |
| Creatinine clearance concomitant with cefepime TDM (mL/min); median [IQR] | | 125 [118-136] | | | 129 [116-137.5] | 121.5 [118-132] | | 0.6 |

N = number ; IQR = interquartile range ; BMI = Body Mass Index ; ASA = American Society of Anesthesiologists ; SAPS II = Simplified Acute Physiology Score II ; SOFA = Sequential Organ Failure Assessment ; VAP = ventilator associated pneumonia ; TDM = Therapeutic Drug Monitoring.

Supplementary AFigure 6: Receiver Operating Characteristic (ROC) curve for a plasma cefepime concentration threshold in predicting cefepime-induced neurotoxicity in patients with EEG monitoring (n=27).


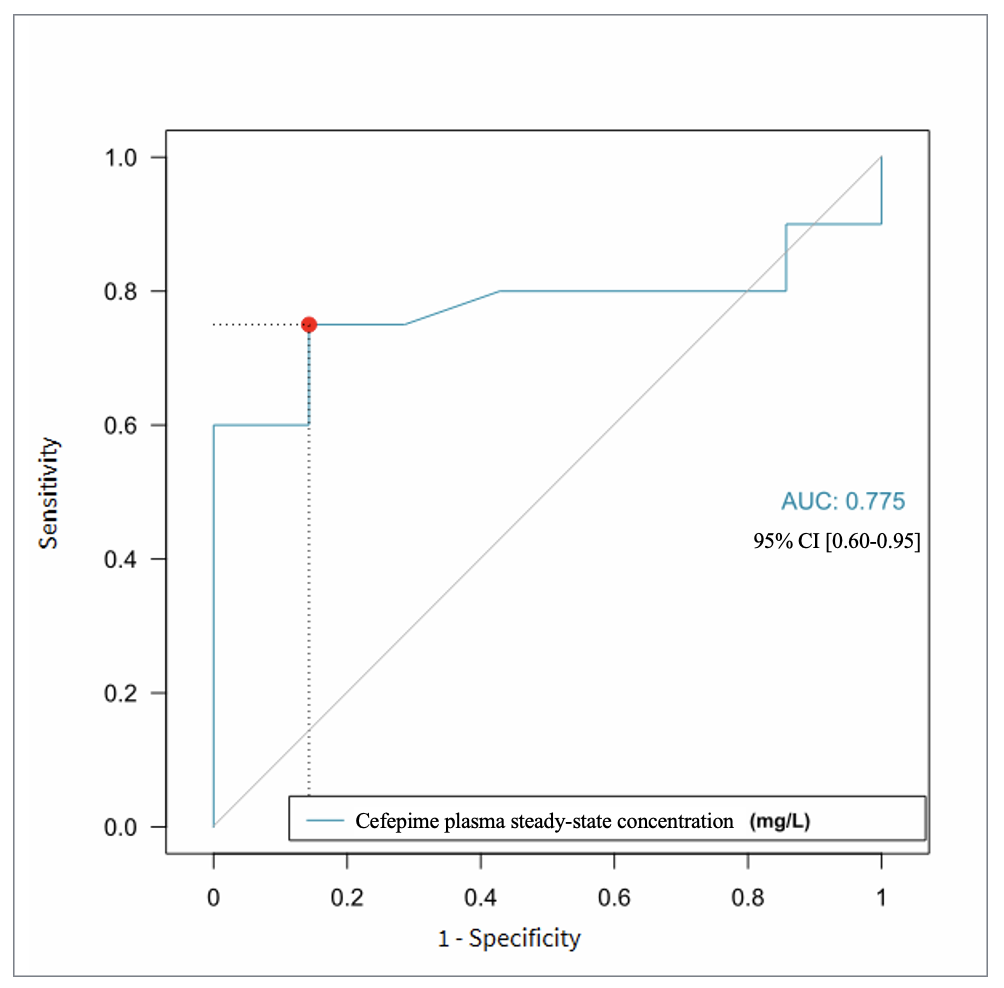


The x-axis represents 1 - specificity and the y-axis represents sensitivity. The grey diagonal line indicates a random chance prediction. The blue curve illustrates the performance of cefepime plasma steady-state concentration in discriminating CIN. The red dot highlights an optimal cutoff point. The area under the curve (AUC) was 0.77 and 95% CI [0.60-0.95]. The optimal cut-off value determined by the ROC curve was 36.4 mg/L with a sensitivity of 75% and a specificity of 86%.

Supplementary ATable 8: Performance characteristics of different cefepime plasma concentration thresholds for CIN prediction.

| Threshold (mg/L) | Sensibility (%) | Specificity (%) | AUC (area under the curve) | [95% CI] |
| --- | --- | --- | --- | --- |
| 20 | 91% | 18% | 0.55 | [0.49-0.60] |
| 25 | 86% | 36% | 0.61 | [0.54-0.68] |
| 30 | 79% | 48% | 0.64 | [0.56-0.71] |
| 35 | 66% | 77% | 0.76 | [0.65-0-83] |
| 40 | 63% | 79% | 0.71 | [0.63-0.79] |
| 45 | 49% | 86% | 0.67 | [0.59-0.76] |
| 50 | 37% | 92% | 0.65 | [0.57-0.73] |

CI = confidence interval.

Supplementary AFigure 7: Impact of Therapeutic Drug Monitoring results turnaround time in hours on dosage adjustment.


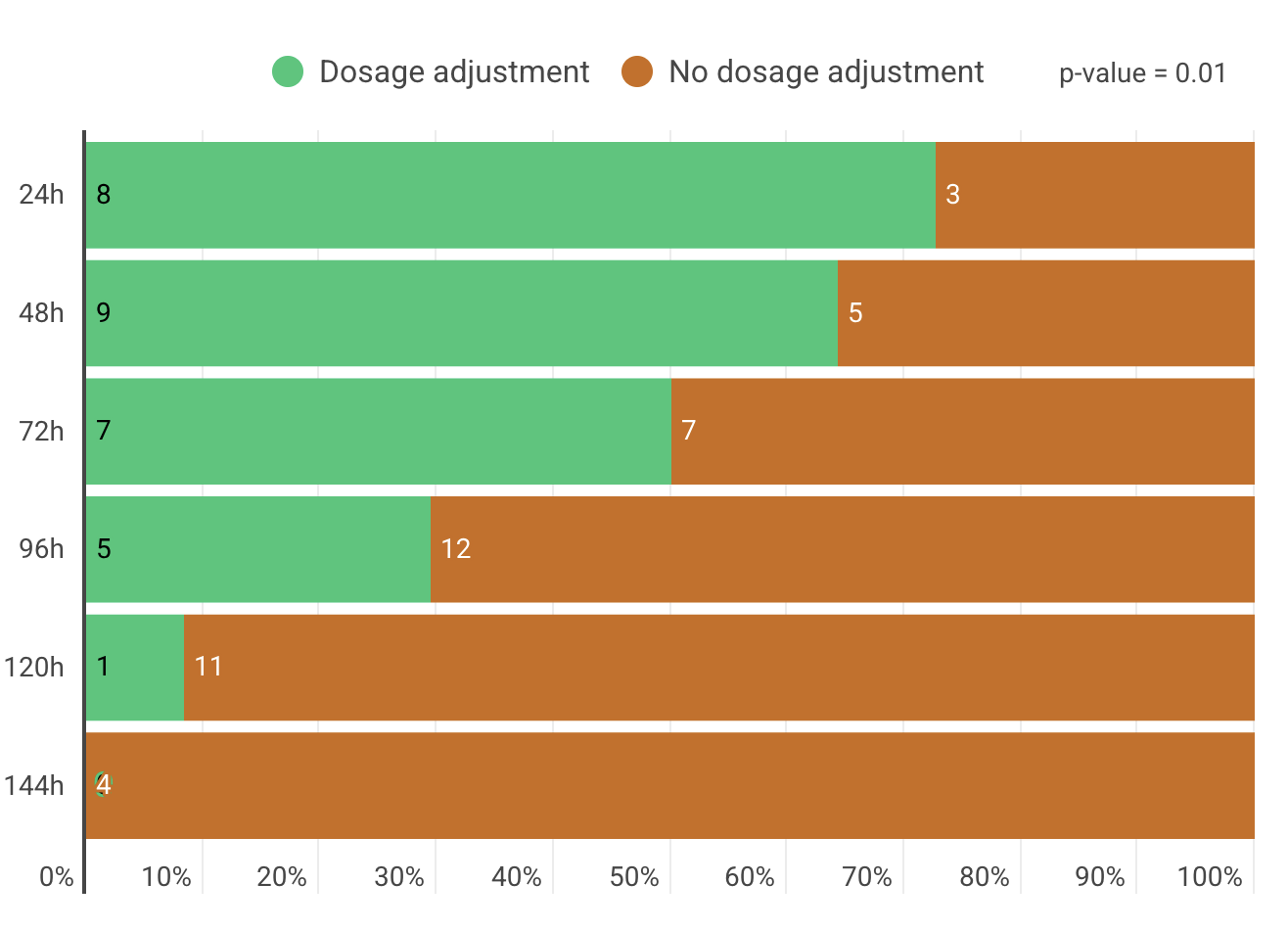


This figure illustrates the proportion of patients who received a cefepime dosage adjustment (green bars) versus those who did not (orange bars), categorized by the time in hours elapsed between cefepime measurement and the availability of the TDM result. The numbers inside the bars indicate the absolute number of patients in each group according to the result turnaround time.

A chi-squared test for trend was used to evaluate the association between the result turnaround time and the likelihood of dosage adjustment, demonstrating a significant decreasing trend over time (p = 0.01).

Supplementary AFigure 8 : The temporal sequence of the 30 patients with a dosage adjustment.

**A.**

**
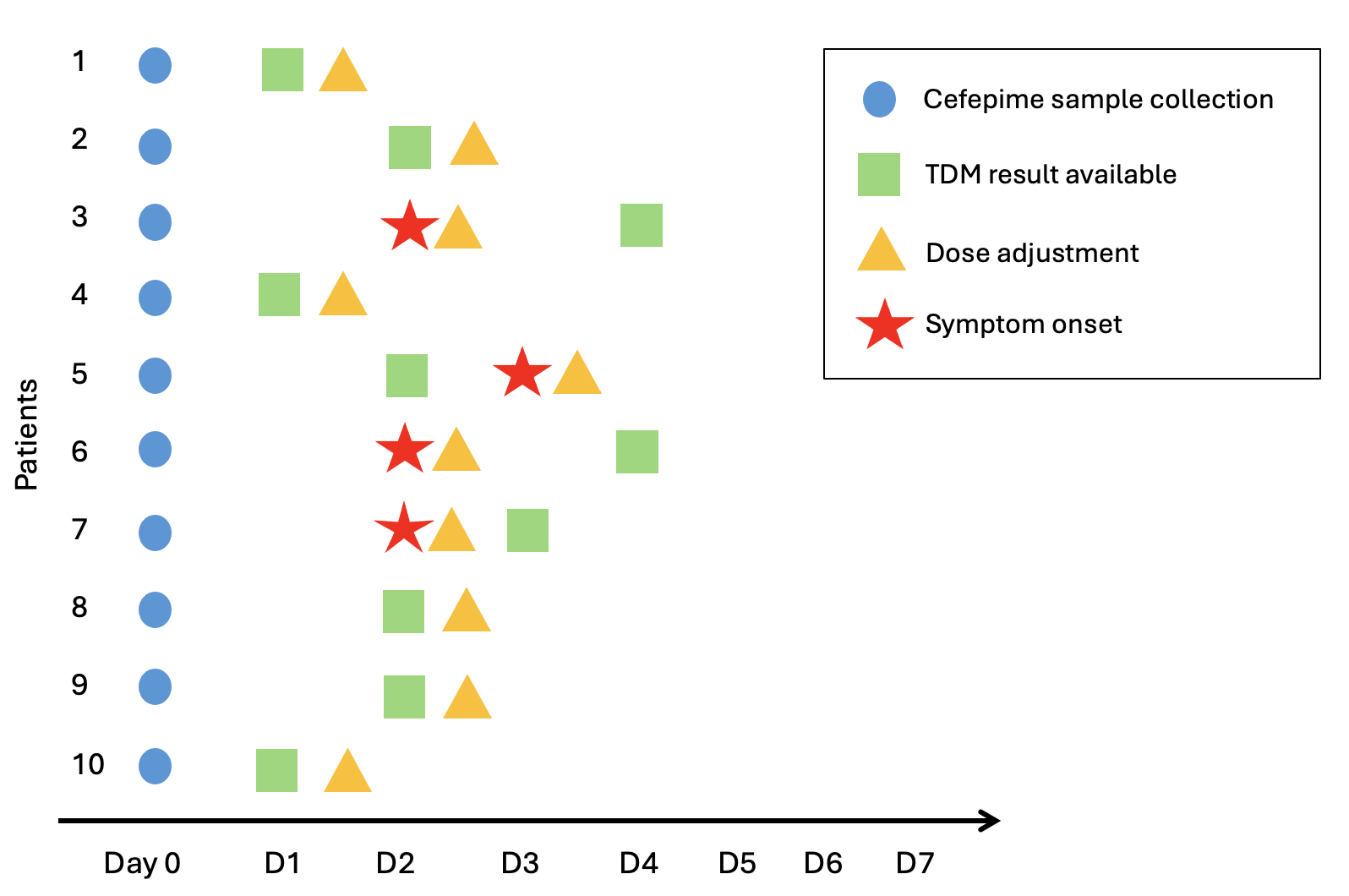
**

**B.**

**
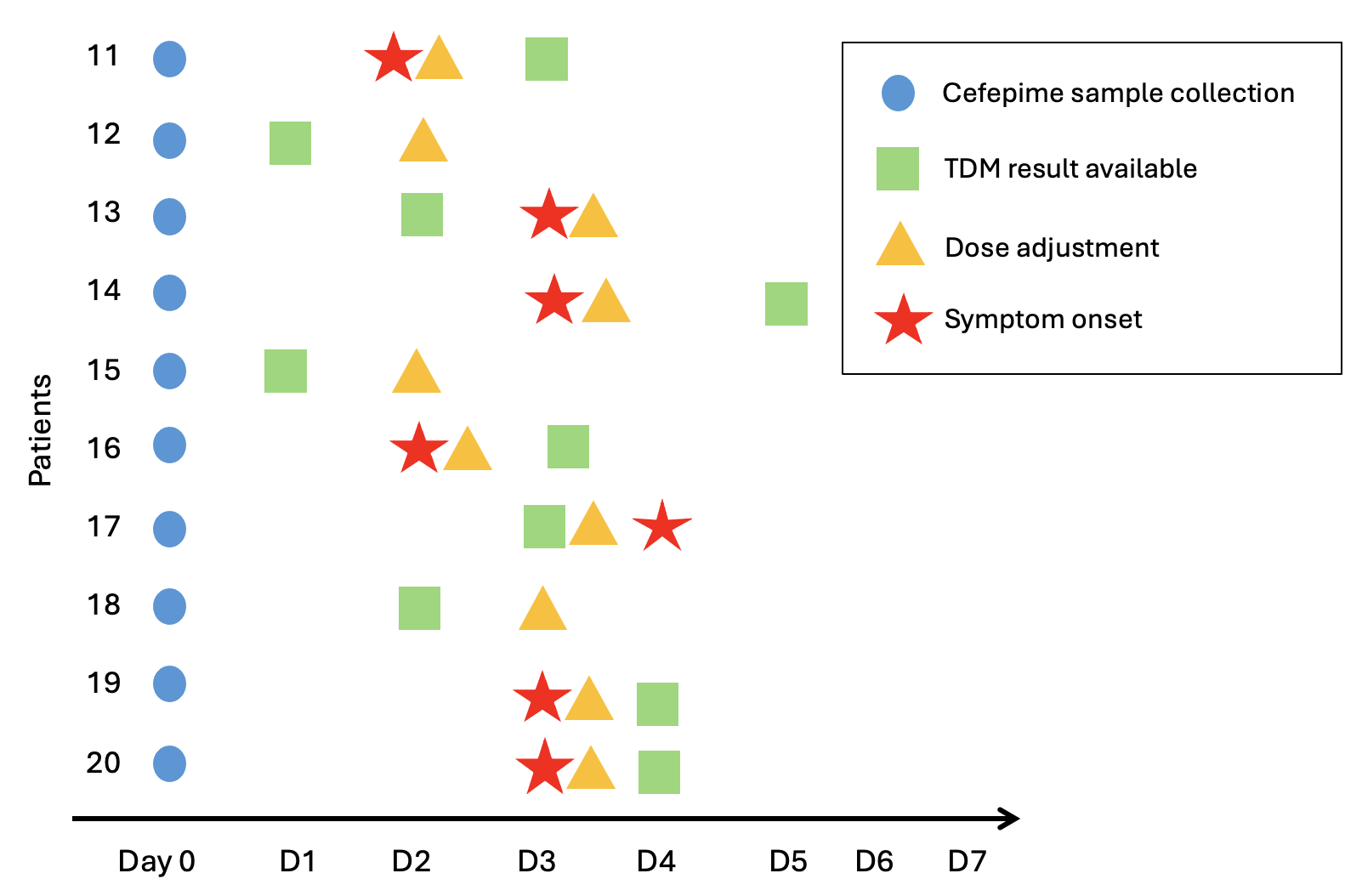
**

**C.**

**
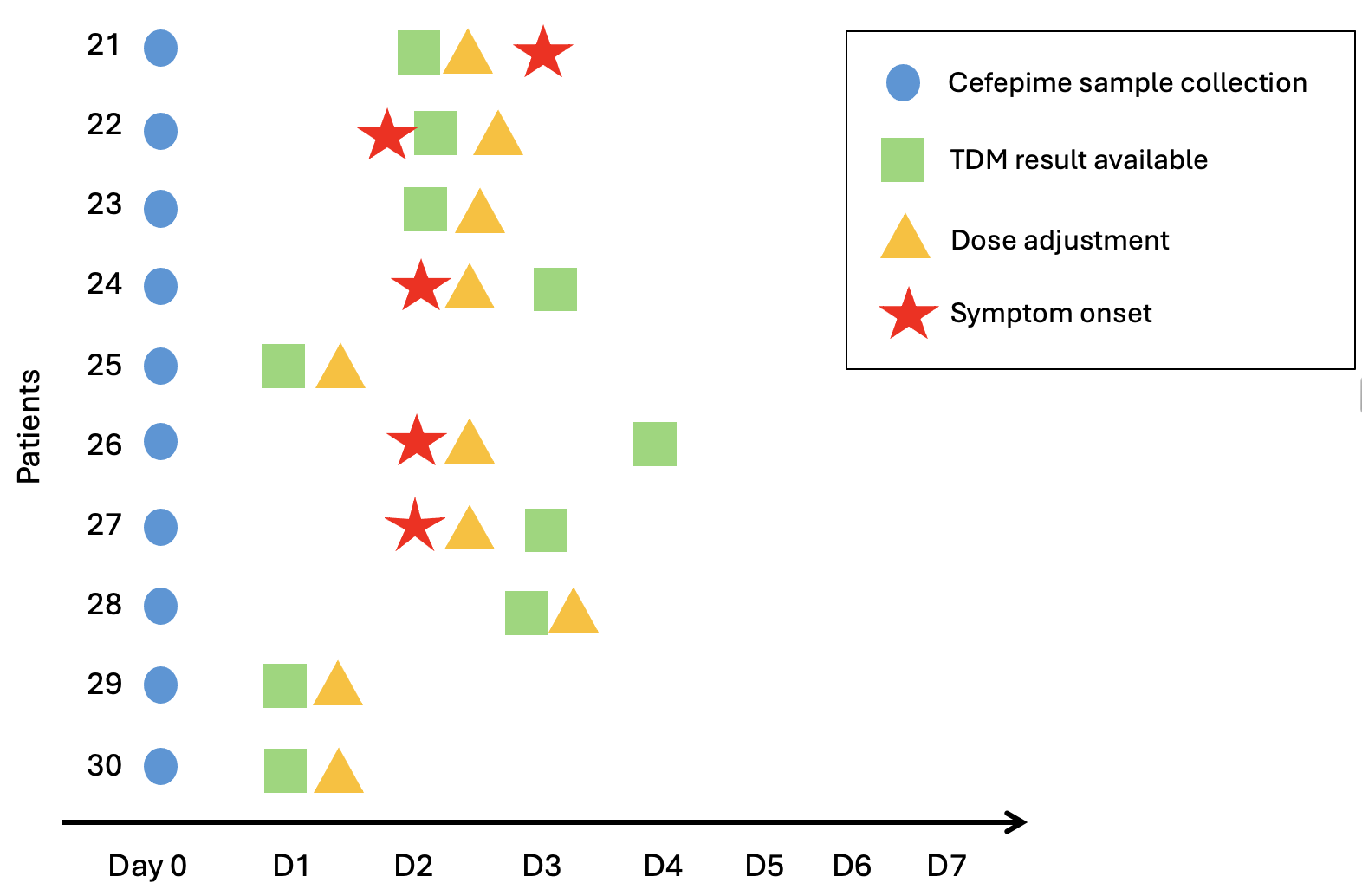
**

D = day

Figure A represents the first 10 patients, figure B represents the subsequent 10 and figure C represents the final 10 patients.

Each symbol represents an event. Given that several sequential events occurred on the same calendar day, the corresponding symbols are closely clustered. These symbols are nonetheless positioned in their strict chronological order to accurately represent the sequence of clinical decision-making and symptom onset.

Fourteen patients had a dosage adjustment based on the TDM results without symptoms of CIN.

Among the patients who presented with symptoms:

- Twelve patients had their dosage modified *following* the onset of symptoms, with the TDM results only becoming available *after* the dosage adjustment.
- For two patients, the TDM results were available *before* the onset of symptoms, but it was the symptoms that triggered the subsequent dosage modification.
- Two patients received a dosage adjustment based on the TDM result, which was then *followed by* the onset of symptoms.
